# Detection of a novel, primate-specific ‘kill switch’ tumor suppression mechanism that may fundamentally control cancer risk in humans: an unexpected twist in the basic biology of TP53

**DOI:** 10.1530/ERC-18-0241

**Published:** 2018-06-25

**Authors:** Jonathan W Nyce

**Affiliations:** ACGT BiotechnologyCollegeville, Pennsylvania, USA

**Keywords:** endocrine-related cancer, endocrine system, p53, TP53, PTEN, tumor suppression, DHEAS, glucose-6-phosphate dehydrogenase, aging and cancer, animal models of cancer, p53-knockout mouse

## Abstract

The activation of TP53 is well known to exert tumor suppressive effects. We have detected a *primate-specific* adrenal androgen-mediated tumor suppression system in which circulating DHEAS is converted to DHEA specifically in cells in which TP53 has been *inactivated*. DHEA is an *uncompetitive* inhibitor of glucose-6-phosphate dehydrogenase (G6PD), an enzyme indispensable for maintaining reactive oxygen species within limits survivable by the cell. Uncompetitive inhibition is otherwise unknown in natural systems because it becomes *irreversible* in the presence of high concentrations of substrate and inhibitor. In addition to primate-specific circulating DHEAS, a unique, primate-specific sequence motif that disables an activating regulatory site in the glucose-6-phosphatase (G6PC) promoter was also required to enable function of this previously unrecognized tumor suppression system. In human somatic cells, loss of TP53 thus triggers activation of DHEAS transport proteins and steroid sulfatase, which converts circulating DHEAS into intracellular DHEA, and hexokinase which increases glucose-6-phosphate substrate concentration. The triggering of these enzymes in the TP53-affected cell combines with the primate-specific G6PC promoter sequence motif that enables G6P substrate accumulation, driving uncompetitive inhibition of G6PD to irreversibility and ROS-mediated cell death. By this catastrophic ‘kill switch’ mechanism, TP53 mutations are effectively prevented from initiating tumorigenesis in the somatic cells of humans, the primate with the highest peak levels of circulating DHEAS. TP53 mutations in human tumors therefore represent fossils of kill switch failure resulting from an age-related decline in circulating DHEAS, a potentially reversible artifact of hominid evolution.

## Introduction

At 38.4%, the human lifetime risk of developing malignant cancer (Ahmad *et al.* 2015; https://www.cancer.gov/about-cancer/understanding/statistics) is one of the highest in the animal kingdom. However, cancer risk is not spread out in a uniform manner over the entire human life span. Rather, cancer risk is extremely low in young humans and increases exponentially as we age. This age-associated increase in cancer risk observed in our species has been thought to reflect the inescapable accumulation of DNA damage experienced over the human life span. A closer examination of the record indicates that in most species, cancer risk remains low and relatively flat throughout their life spans, even in animals such as elephants that have lifespans as long as ours (Buffenstein 2008, Fang *et al.* 2014, Abegglen *et al.* 2015, Sulak *et al.* 2016). For example, a study of animals dying at the San Diego Zoological Gardens demonstrated that neoplasia was present at necropsy in 2.75% of 3127 mammals, 1.89% of 5957 birds, and 2.19% of 1233 reptiles (Effron *et al.* 1977). A report from the Royal Zoological Gardens of Amsterdam described a tumor incidence of zero for fifty autopsied primates, and 5.7% for 35 autopsied carnivores (Borst *et al.* 1972). An older study of 5365 necropsies of mammals and birds at the Philadelphia Zoological Gardens demonstrated an overall incidence of neoplasia of about 2% (Ratcliffe 1933). Lombard & Witte (1959), also using data acquired at the Philadelphia Zoological Gardens, reported a tumor incidence of 1.59% in 754 circopithecidaen primates, and in a study at the Yerkes Primate Center, only six of 1066 primates subjected to thorough postmortem autopsy demonstrated malignant cancer (McClure 1973). A recent large, multi-institutional study confirmed these earlier works in large measure, demonstrating that cancer risk in most long-lived animals is low (2–6%) and independent of life span (Abegglen *et al.* 2015). Cancer risk as a function of increasing age in elephants, wildebeest, moose and most other long-lived animals is thus linear, with little increase in slope with advancing age. This is in sharp contrast to cancer risk in humans, which increases in conformance with a logistic curve with a 30-year lag phase followed by steep exponential kinetics until very late in the life span. Taken together, these observations suggest that tumor suppression mechanisms in non-human species are generally of a type that does not substantially diminish over their lifespan, whereas those in humans do diminish with increasing age. The much lower cancer rate in other long-lived species also indicates that, when tumor suppression systems function throughout life, while most kinds of genomic damage may accumulate, that subclass of damage that would initiate tumorigenesis is efficiently extinguished.

The p53 tumor suppressor is an ancient protein found in organisms ranging from *Caenorhabditis elegans* to *Homo sapiens* (Derry *et al.* 2001). Because of its intimate role in countering neoplastic transformation in multi-cellular animals, p53 has been dubbed ‘the guardian of the genome’. Over the past four decades, a paradigm has evolved in which p53 is thought to function in a very similar manner across widely disparate species. According to this paradigm, DNA damage activates the transcription factor properties of p53, such that DNA replication is halted until the damaged DNA can be repaired. If the damage is too great, p53 induces apoptosis by activation of an alternative pathway (for reviews, Schumacher *et al.* 2001, Kastenhuber & Lowe 2017, Yue *et al.* 2017). More than half of all human tumors have been found to have mutations in *TP53* (the human version of *p53*), and TP53 appears to be inactivated by other means in the remaining tumors where such mutations are absent (Hollstein *et al.* 1991, Petitjean *et al.* 2007, Olivier *et al.* 2010, Merkel *et al.* 2017). Mutations in the *p53* gene are also prevalent in spontaneous tumors of dogs and cattle, species in which monitoring of neoplasia is routine, and *p53* mutations in these species occur in the same ‘hot spots’ as in human tumors (Zhuang *et al.* 1997, Loukopoulos *et al.* 2003). Further support for the paradigm of a universal mechanism of action for p53 in mammalian cancer came from the finding that humans with germline mutations in* TP53* experience an inordinately high risk of a wide array of tumor types before the age of 30 years (Varley 2003, Guha & Malkin 2017) and that inactivation of p53 in the so-called p53-knockout mouse duplicates this high risk of a wide array of tumor types occurring at an early age (Lozano & Liu 1998, Kenzelmann Broz & Attardi 2010). These findings have encouraged an exceptional degree of confidence among workers in the field that mouse models of tumor suppression offer reasonable approximations of mechanisms of tumor suppression in humans. Thus, for the past several decades, the guiding paradigm with respect to the p53 tumor suppressor has been that it functions in a more or less similar manner across species at least as diverse as man and mouse, and probably across species even more diverse than that. It is our belief, however, that the establishment of this paradigm has come at the expense of ignoring more fundamental paradigms associated with mechanisms of speciation. In this commentary, we discuss our deep reservations with the prevailing p53 paradigm, point out important ways in which it may have misled the endeavor of cancer research, both basic and clinical, and offer an alternative viewpoint based upon new discoveries in species-specific mechanisms of tumor suppression.

### Species-specific mechanisms of tumor suppression challenge the prevailing p53 paradigm

The concept of species-specific mechanisms of tumor suppression is gaining increasing support (Contente *et al.* 2003, Wang *et al.* 2007, Leroi *et al.* 2013, Heyne *et al.* 2015, Tollis *et al.* 2017, Zhou *et al.* 2017). Recent evidence in the elephant (Abegglen *et al.* 2015, Sulak *et al.* 2016), the naked mole rat (Buffenstein 2008, Fang *et al.* 2014), the blind mole rat (Avivi *et al.* 2007, Shams *et al.* 2013) and canines (Nyce 2017), all support the concept that species-specific mechanisms of tumor suppression may in fact be relatively common. This should not be too surprising, since every species’ evolution through spacetime is unique. The very concept of species entails variations on the themes of body size, lifespan, metabolic rate, reproductive rate, environmental niche and physical and biochemical adaptations to exploit that environmental niche, each of which can be expected to influence risk of neoplastic transformation. By presuming a universal mechanism of action for p53, the prevailing paradigm ignores the fact that *all* enabling elements of a species’ forward movement through spacetime represent variables that are under integrated selection to maximize exploitation of environmental resources *and to simultaneously minimize opposing forces, such as neoplastic transformation*. It thus stands to reason that mechanisms of tumor suppression may evolve that incorporate features unique to a particular species, particularly in longer-lived and larger animals. The current paradigm of universal mechanisms of tumor suppression that are independent of species therefore appears to be incorrect and may have led us quite far down an unproductive path. For example, *Mus musculus* and *Rattus norvegicus* were selected as model systems for the study of human cancer precisely because they were small, had short, accelerated life spans and had a high reproductive rate – exactly the features that, in hind sight, would be expected to make them species-specific models of murine, not human, cancer. To put this in the sharpest possible relief, murine species use small size and short lifespan as mechanisms to maximize exploitation of their environment while simultaneously minimizing neoplastic transformation. Small size minimizes the number of stem cells at risk for neoplastic transformation, and short lifespan resets accrued mutations to near zero at very short intervals in successive generations, spreading risk across time. This murine strategy is very efficient in that it requires only the canonical p53 repertoire already so well analyzed using p53-knockout mice. The prevailing p53 paradigm thus appears to provide accurate descriptions of this minimalist approach to tumor suppression taken by small, short-lived animals such as mice. As we shall discuss, it is a completely different tumor suppression strategy than those that evolved in larger, longer-lived species such as humans, elephants and whales – the strategies of which will clearly be as different from each other as they are from mice because of the different environments they exploit, and the species-specific mechanisms that have evolved to enable exploitation of those environments. Such species could only evolve large bodies and long lifespans by augmenting the canonical p53 repertoire in ways that are frequently specific to their lineage and sometimes specific to their species. Such considerations, and our identification of a primate-specific adrenal androgen-mediated tumor suppression system dependent upon circulating DHEAS – which does not occur in murine species – quite strongly suggest that data provided by mouse and rat models are applicable only to those species and are completely incapable of meaningful translation to human cancer. We are not the first to make this observation:

"The history of cancer research has been a history of curing cancer in the mouse. We have cured mice of cancer for decades – and it simply didn’t work in humans."

– Dr Richard Klausner

Former Director of the National Cancer Institute (Cimons *et al.* 1998)

### Identification of an Anthropoid primate-specific kill switch tumor suppression system

Exposure to significant cellular stress is well known to activate the p53 tumor suppressor to induce apoptosis by both transcription-dependent and transcription-independent mechanisms (Speidel 2010, Le Pen *et al.* 2016, Castrogiovanni *et al.* 2017). We have recently reported our detection in canines of a rudimentary form of an otherwise primate-specific adrenal androgen-mediated ‘kill switch’ in which cell death is triggered by the *inactivation* of p53 (Nyce 2017). By analogy with other long-lived animals such as the elephant, this adrenal androgen-mediated kill switch mechanism may represent the primary means of defense used by our species to prevent transformation caused by genotoxins. It has been hiding in plain sight within the p53 repertoire and may have kept so well hidden because it depends on the unique, primate-specific evolution of extraordinarily high post-natal levels of circulating DHEAS. In humans, this begins at about age 6 years with the advent of adrenarche – the development of the adrenal *zona reticularis*, a tissue the only apparent function of which is to synthesize DHEAS in extremely large amounts and secrete it into the bloodstream (Bird 2012, Rege & Rainey 2012). While both Anthropoid primates (humans, chimpanzees, bonobos, gorillas, etc.) and Strepsirrhine primates (lemurs) have circulating DHEAS, such levels are orders of magnitude higher in Anthropoid as compared to Strepsirrhine primates, and true adrenarche may only occur in the human, chimpanzee and bonobo (Nakamura *et al.* 2009, Behringer *et al.* 2012). Nevertheless, dogs have a rudimentary *zona reticularis* and a homologue of adrenarche has been reported in them (Schiebinger *et al.* 1981, Perez-Fernandez *et al.* 1987). Based upon this finding, we formulated the hypothesis that canines might also possess a homologue of the otherwise primate-specific adrenal androgen-mediated tumor suppressor system and that at least some canine tumors might retain sensitivity to triggering of this system. Indeed, certain canine tumors do respond to triggering of the kill switch in a manner that has never, to our knowledge, been observed in murine models (Nyce 2017).

Circulating DHEAS does not occur in common laboratory rats or mice, and the near exclusive use of such rodent models in cancer research over the past 40 years clearly contributed to the delay in the discovery of the primate-specific, adrenal androgen-mediated kill switch tumor suppression system. Additional research impediments have also contributed to the kill switch mechanism remaining occult throughout these decades of p53 research. Thus, it cannot be studied in transformed cells, because these have already escaped succumbing to it because of kill switch failure (see below); following such failure, such transformed cells have also incurred an obfuscating patchwork of follow-on mutations and epigenetic variations. The kill switch tumor suppressor system is also a single cell phenomenon, and single cell analysis techniques have not yet reached the level of sophistication required to detect in real time a unique event occurring in a vast excess of unaffected cells at an approximate rate of 2 × 10^−7^; let alone an event designed to extinguish that cell from existence. Our detection of this kill switch tumor suppression mechanism depended upon a rudimentary form of it occurring in dogs, and the fact that our laboratory works exclusively with dogs with spontaneous cancer (Nyce 2017).

### The mechanics of the kill switch

DHEAS and DHEA represent the Dr Jekyll and Mr Hyde of androgen biology. DHEAS can circulate at very high levels without toxicity because, as a hydrophilic anion, it requires active transport into the cell and, as long as it remains in its sulfated form, it exerts no untoward effects upon intermediary metabolism. DHEA, on the other hand, is lipophilic, freely diffuses into cells, and is a potent *uncompetitive* inhibitor of the critical enzyme glucose-6-phosphate dehydrogenase (G6PD). Circulating DHEA must therefore be maintained at very low serum concentrations, orders of magnitude below its inhibition constant for G6PD (*K*_i_ = 18.5 μM; compare DHEAS *K*_i_ = 310 μM (Gordon *et al.* 1986); peak serum concentrations of DHEA of ≈ 30 nM, and of DHEAS of ≈ 11.5 µM (Labrie *et al.* 1997)). Because of its extreme rarity, the mechanics of uncompetitive inhibition are frequently ignored. Uncompetitive inhibition requires that the substrate first binds to the enzyme, forming an enzyme:substrate complex (ES) that flexes the enzyme, creating a binding site for the inhibitor. This creates enzyme kinetics in which inhibitor binding uniquely decreases both *K*_m_ and *V*_max_. While all mechanisms of enzyme inhibition increase substrate concentration, only in uncompetitive inhibition does the increase in substrate concentration enhance enzyme inhibition rather than suppress it, by increasing the amount of ES to which the inhibitor can bind. Thus, in the presence of high intracellular concentrations of substrate and inhibitor, uncompetitive inhibition becomes irreversible. This is modeled by the equation:

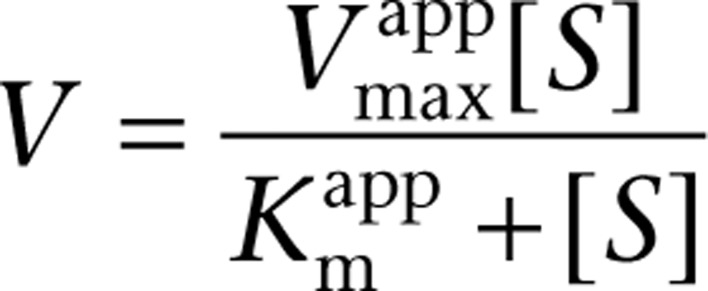



Where 
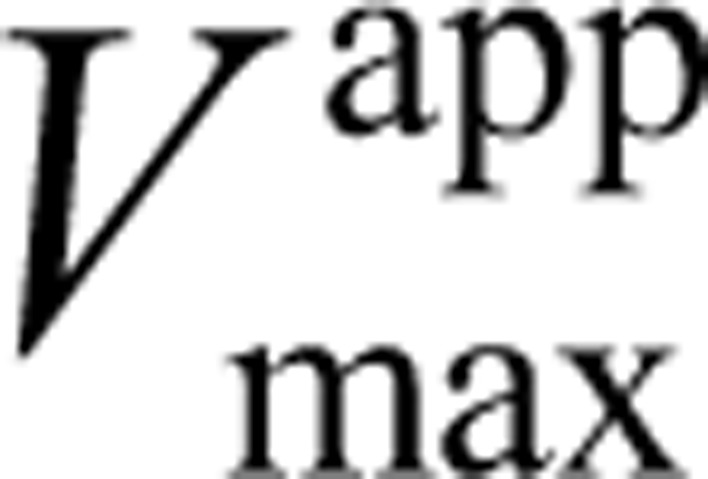
 is the apparent *V*_max_ given by:

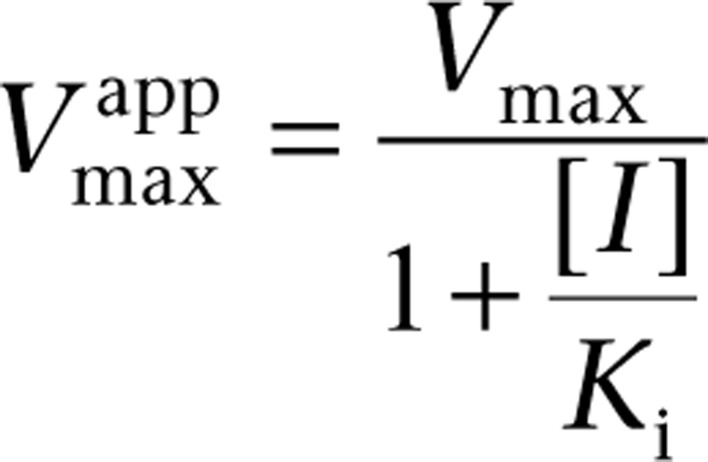




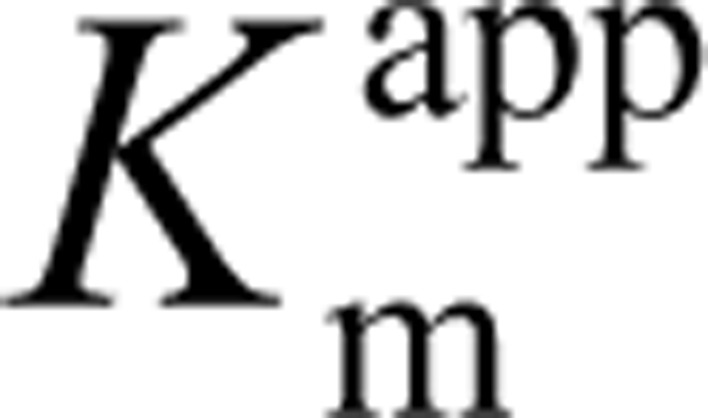
 is the apparent *K*_m_ given by:

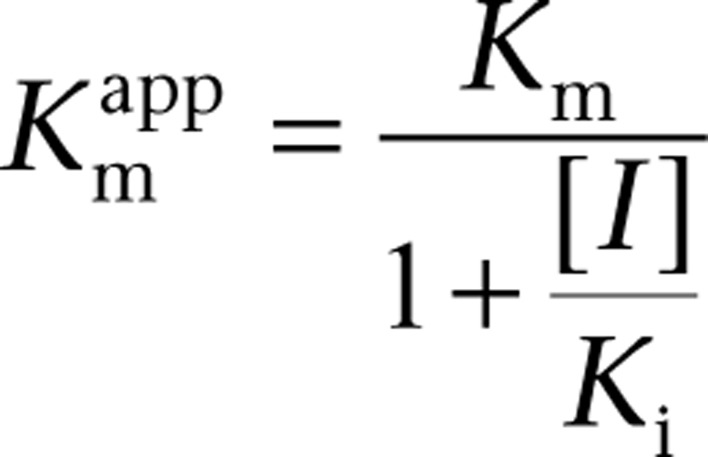



As noted by Cornish-Bowden (1986), the potential of uncompetitive inhibitors to induce catastrophic toxicity has made them almost nonexistent in natural systems:

"Cases of uncompetitive inhibition by species that are not involved in the reaction are virtually unknown … Uncompetitive effects may not merely be mechanistically implausible but may be so detrimental to organisms that display them that there has been evolutionary selection against such inhibition by naturally occurring metabolites. *It may therefore be worthwhile to point out that any metabolic pathway in which uncompetitive inhibition can occur can potentially respond catastrophically to the presence of inhibitor*."

**Figure 1:**
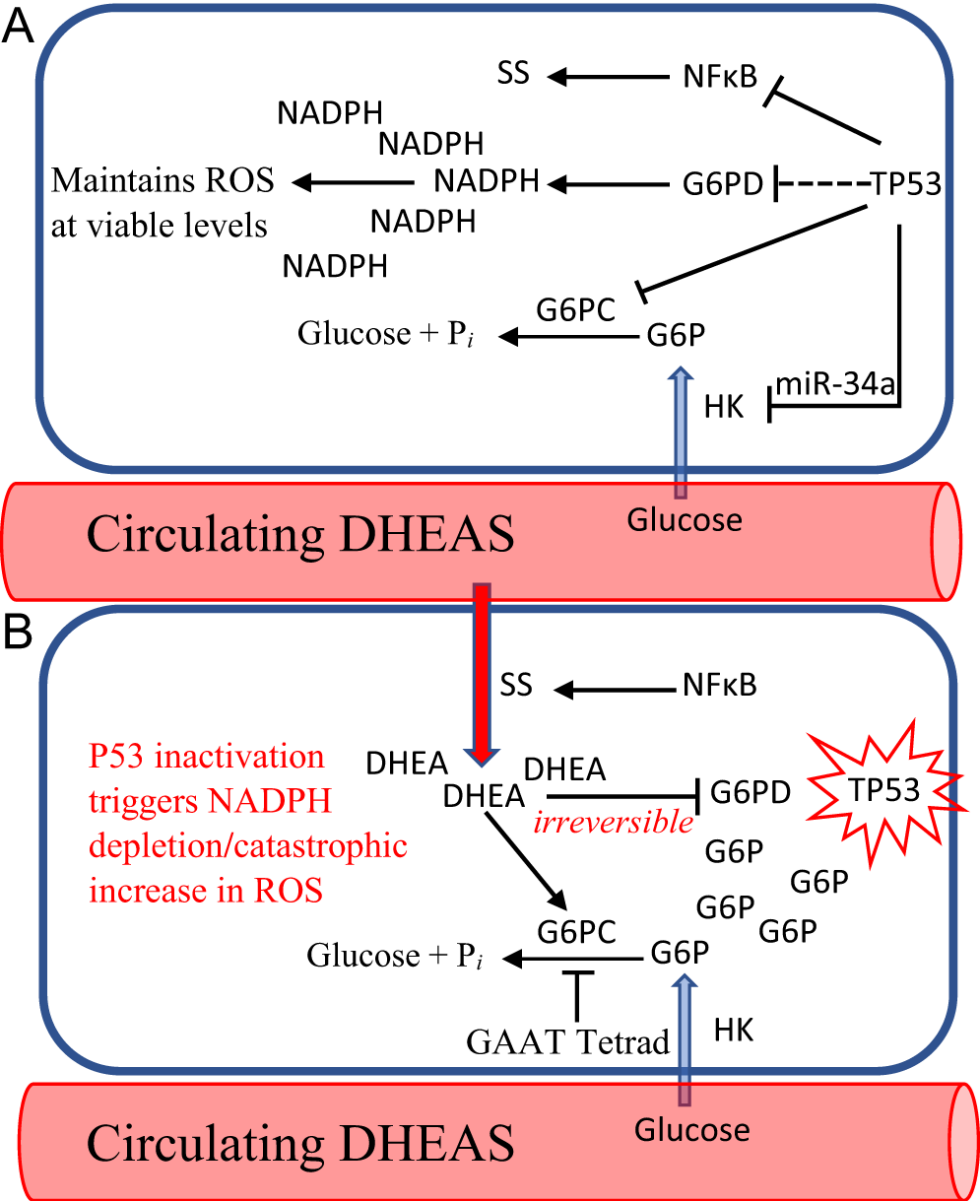
Mechanism of action of the adrenal androgen-mediated kill switch tumor suppression system. (A) Cell with normal p53 function. (B) A somatic cell in which mutation of p53 has occurred. Cells with inactivated p53 act as a sink for circulating DHEAS, which is imported into the cell by OATPs (downward arrow). In addition to high circulating levels of DHEAS, an Anthropoid primate-specific sequence motif (GAAT) in the G6PC promoter was also required to enable kill switch function. A full colour version of this figure is available at https://doi.org/10.1530/ERC-18-0241.

Among several other critical cellular duties, G6PD supplies the NADPH required to maintain ROS concentrations at levels that are survivable for the cell (Yang *et al.* 2016, Hq *et al.* 2017). If the conditions for irreversible uncompetitive inhibition of G6PD are met in a cell, the resulting depletion of intracellular NADPH will lead to a rapid, catastrophic increase in intracellular ROS in that cell. The triggering of such irreversible uncompetitive inhibition of G6PD in cells affected by TP53 inactivation occurs by a series of well-described reactions (Fig. 1). Thus, inactivation of TP53 de-represses Glut 1 and Glut 4 transporters (Schwartzenberg-Bar-Yoseph *et al.* 2004, Shen *et al.* 2012), bringing excessive amounts of glucose into the injured cell. Inactivation of TP53 also de-represses hexokinase-1 and -2 by eliminating miR-34a (Kim *et al.* 2013), increasing intracellular pools of glucose-6-phosphate (G6P). Excess G6P binds to G6PD, creating binding sites for the small amount of intracellular DHEA that originally exists in the cell. G6PD E:S then acts as a sink for DHEA, stimulating OATP2B1, the transport protein responsible for importing DHEAS into the cell. Inactivation of TP53 also hyperactivates NFKB (Weisz *et al.* 2007, Kawauchi *et al.* 2008, Cooks *et al.* 2013), triggering steroid sulfatase (Hattori *et al.* 2012, Dias & Selcer 2016), which potentiates the importation of DHEAS and its intracellular activation to DHEA. OATP2B1 is also stimulated by the intracellular presence of de-sulfated androgens (Grube *et al.* 2006), such that as the intracellular concentration of DHEA rises, DHEAS is imported into the p53-affected cell at an ever-accelerating rate. With all limits upon their synthesis eliminated, intracellular concentrations of G6P and DHEA quickly rise, causing irreversible uncompetitive inhibition of G6PD, complete depletion of intracellular NADPH and consequent catastrophic increase in intracellular ROS in the TP53-affected cell. It is important to point out that loss of NADPH eliminates redox control of intracellular ROS both by depletion of reductant required for the function of redox proteins, *and* by inhibition of the synthesis of those same redox proteins. Thus, HMG CoA reductase is an unusual enzyme in intermediary metabolism in that it requires two moles of NADPH for each mole of mevalonate produced. It is therefore extremely sensitive to NADPH depletion (Schulz & Nyce 1991). We have previously demonstrated that DHEA sufficient to deplete intracellular NADPH and inhibit HMG CoA reductase blocks the isoprenylation of the RAS oncoprotein, as well as other mevalonate-dependent pathways (Schulz & Nyce 1991). These additional mevalonate-dependent pathways include the synthesis of selenoproteins such as thioredoxin reductase (TRX) and glutathione peroxidase (GPX), the translation of which require mevalonate-dependent N6-isopentenyladenosine in selenocysteine tRNA [Ser]Sec (Warner *et al.* 2000). Such inhibition of selenoprotein synthesis in cells in which uncompetitive inhibition of G6PD has become irreversible is likely to constitute a major component of the kill switch mechanism because, of the 25 known human selenoproteins, more than half are involved in the control of the cellular redox environment (Mangiapane *et al.* 2014). TRX and GPX are unconditionally essential to redox control, and their function would be particularly degraded because in addition to their physical depletion due to inhibition of their synthesis caused by NADPH cofactor depletion of HMG CoA reductase in the mevalonate pathway, they also require NADPH for regeneration of their reduced states at their site of action.

This kill switch mechanism, which targets cancer at the level of the initial potentially tumorigenic cell – a point in tumorigenesis that we refer to as the singularity – may prevent most TP53 mutation events from initiating tumorigenesis when it is acting at optimal efficiency. The kill switch mechanism offers the first explanation of why there would exist an uncompetitive, potentially irreversible inhibitor of a critical enzyme such as G6PD.

According to this model, human tumors with TP53 mutations represent instances in which this kill switch mechanism had failed to be triggered, for example, because of inadequate amounts of circulating DHEAS in aging modern humans (see below). TP53 mutations in human tumors are thus fossils of such kill switch failure. We have demonstrated that in some spontaneous tumors, appropriate manipulation of the adrenal androgen system can trigger a failed kill switch to fire, resulting in tumor regression (Nyce 2017). This finding provided indirect evidence for the primate-specific nature of the kill switch, as it is only observed in species with circulating DHEAS, which consists almost exclusively of Anthropoid primates, of which humans have by far the highest peak levels. Quite direct evidence for the kill switch mechanism comes from Anthropoid primate-specific genetic modifications that were required to enable kill switch function.

### Genetic evidence supporting the species-specific evolution of the adrenal androgen ‘kill switch’

If the DHEAS-mediated kill switch evolved in a species-specific manner – in humans, but not in mice and rats – is there evidence for this species specificity in the genetic record? In addition to primate-specific circulating DHEAS, additional changes in intermediary metabolism were required to enable the kill switch to function in humans but not in mice or rats. For example, irreversible uncompetitive inhibition of G6PD requires glucose-6-phosphate (G6P) substrate to accumulate to high intracellular concentrations. This is not possible if the enzyme glucose-6-phosphatase (G6PC) is active, because G6PC catabolizes G6P to glucose and inorganic phosphate, which would prevent the accumulation of G6P. While primarily thought of as an hepatic enzyme that plays a major role in glucose homeostasis, G6PC is known to be dysregulated in an array of human tumor types (Abbadi *et al.* 2014, Guo *et al.* 2015) and is a target of p53 regulation (Kim *et al.* 2013, Zhang *et al.* 2014).

G6PC activity is modulated by peroxisome proliferator-activated receptor gamma coactivator 1-alpha (PGC-1alpha), an important regulator of energy expenditure. PGC-1alpha directs hepatic nuclear factor-4alpha (HNF-4alpha), a member of the steroid/thyroid hormone receptor superfamily, to a specific dodecanucleotide activating regulatory site in the G6PC promoter. PGC-1alpha is potently stimulated by DHEA (Yokokawa *et al.* 2015), and such stimulation would disable kill switch function by removing G6P substrate. In the mouse, the presence of intracellular DHEA induces PGC-1alpha, activating G6PC and preventing the accumulation of G6P. This explains why administration of DHEA to p53^−/−^ mice is not toxic (Perkins *et al.* 1997, Wang *et al.* 1997).

However, something remarkable and telling has occurred in humans. Schilling and her colleagues identified a G6PC promoter sequence motif immediately downstream from the HNF-4alpha-binding site that appears to regulate PGC-1alpha control of G6PC activity (Schilling *et al.* 2008). Whereas in murine species this motif consists of ACAG and is permissive for PGC-1alpha-mediated activation of G6PC activity, in humans, it is GAAT which disables PGC-1alpha-mediated stimulation of G6PC activity. In contrast to rats and mice, then, in humans intracellular DHEA cannot induce PGC-1alpha-mediated activation of G6PC. In a species-specific manner, G6P can accumulate in human cells in the presence of intracellular DHEA, which it cannot do in mouse or rat cells. Species-specific response to intracellular DHEA has been noted before, with normal human and rat aortic vascular smooth muscles cells responding in opposite fashion to DHEA exposure (Yoneyama *et al.* 1997).

**Figure 2:**
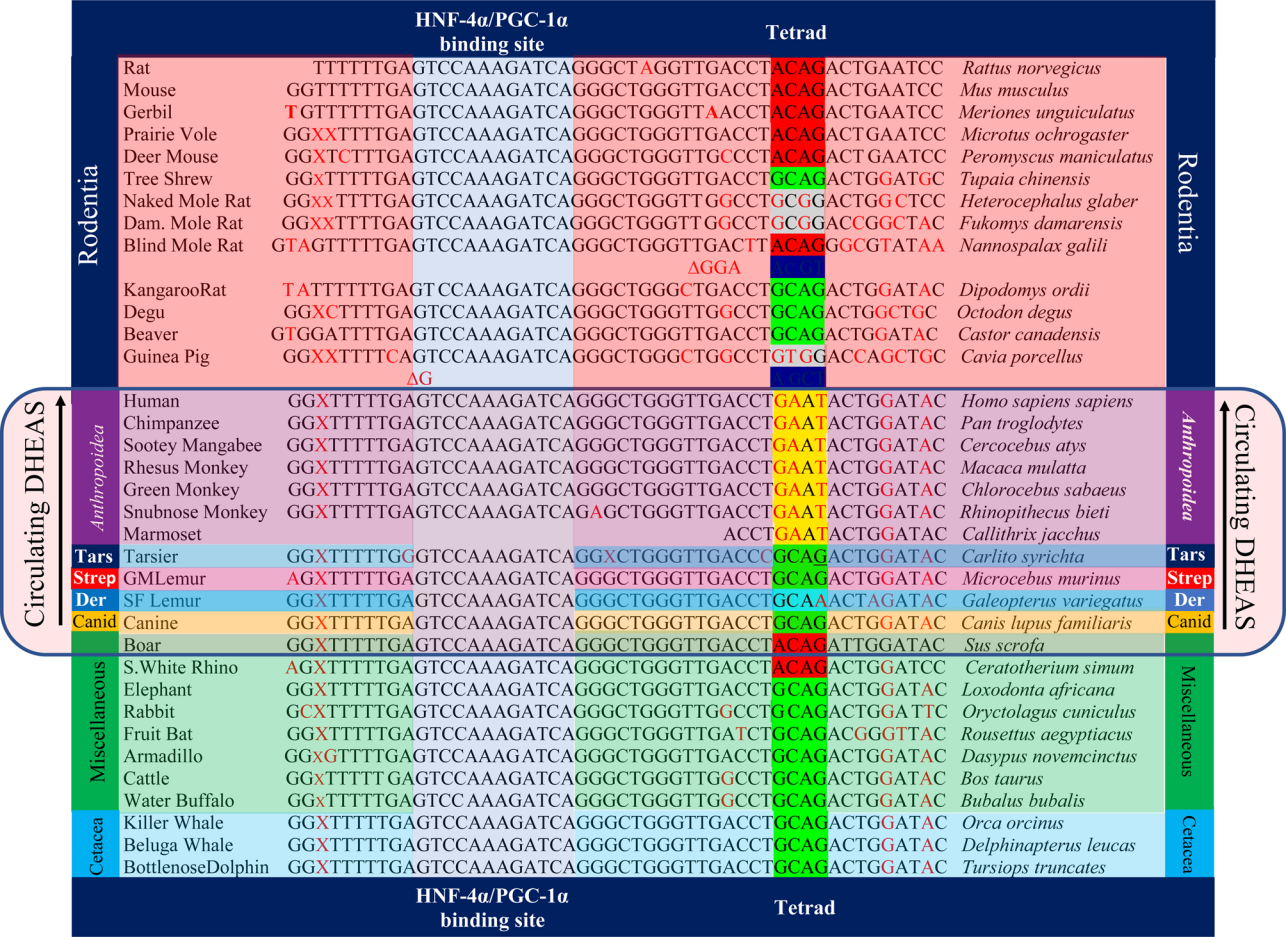
Anthropoid primate-specific modification of G6PC promoter to produce GAAT tetrad enables accumulation of G6P required for catastrophic uncompetitive G6PD inhibition. The highly conserved dodecanucleotide HNF-4alpha/PGC-1alpha binding site is highlighted in vertical blue box. The G6PC promoter tetra-nucleotide that disables (ACAG) or enables (GAAT) accumulation of G6P is labeled tetrad. Accession numbers for listed sequences can be found in Supplementary Section 2. Site-specific insertions are depicted as ∆ followed by the inserted sequence. Tars, tarsiers; Strep, Strepsirrhine primates such as lemurs and lorises; Der, Dermoptera, the closest mammalian order relative to primates.

**Figure 3:**
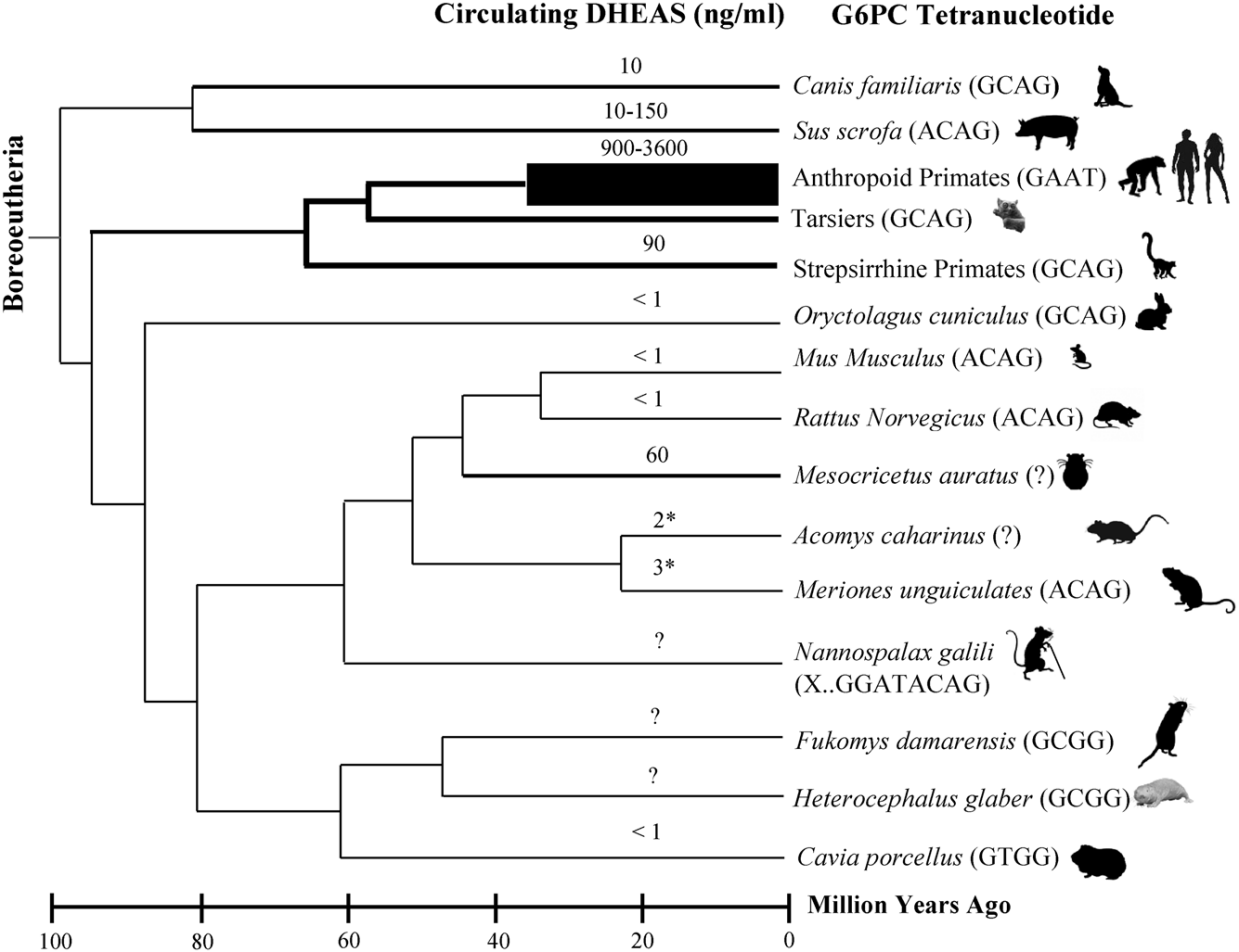
High levels of circulating DHEAS and the G6PC tetranucleotide GAAT enabling kill switch function are specific to the Anthropoid primate lineage, with DHEAS reaching highest levels by far in *Homo sapiens*. Rodent species lack both circulating DHEAS and the G6PC promoter tetranucleotide (GAAT) that permits intracellular G6P to accumulate. *DHEA measured, not DHEAS. Circulating DHEAS shown for canine (Schiebinger *et al.* 1981, Odell & Parker 1985, Tremblay & Belanger 1985, Ashley *et al.* 1988, Mialot *et al.* 1988, Frank *et al.* 2003, Mongillo *et al.* 2014, Rondelli *et al.* 2015); boar (Schuler *et al*. 2014); Anthropoid primates (Cutler *et al.* 1978, Axelson *et al.* 1984, Sulcova *et al.* 1997, Kemnitz *et al.* 2000, Bjornerem *et al.* 2006, Bernstein *et al.* 2012, Blevins *et al.* 2013); Strepsirrhine primates (Perret & Aujord 2005); rabbit (Alexandersen *et al.* 1999); mouse and rat (van Weerden *et al.* 1992); golden hamster (Pieper & Lobocki 2000); spiny mouse (Quinn *et al.* 2013); Mongolian gerbil (Fenske 1986) and Guinea pig (Belanger *et al.* 1989). The evolutionary chronology pictured was redrawn after Kim *et al.* (2017).

We discovered that the GAAT tetranucleotide (Tetrad) that disables PGC-1alpha-mediated activation of G6PC is specific to the Anthropoid primate lineage and does not occur in Strepsirrhine primates (e.g., lemurs) or non-primate species (Fig. 2). Critically, this means that the GAAT Tetrad that disables PGC-1alpha-mediated activation of G6PC *is specific to lineages with high circulating DHEAS*, as lemurs and other Strepsirrhine primates have circulating DHEAS levels that are more than 40-fold less than those observed in Anthropoid primates (Fig. 3). Anthropoid primates, particularly humans, are thus unique among all species in the possession of high levels of circulating DHEAS *and* the G6PC promoter sequence motif that enables the accumulation of G6P in the presence of intracellular DHEA. This provides unambiguous evidence that the ability to induce irreversible uncompetitive inhibition of G6PD in somatic cells is an important aspect of the evolution of Anthropoid primates, culminating in *Homo sapiens*, the Anthropoid species with the highest peak levels of circulating DHEAS. The fact that DHEA and p53 (and PTEN) have co-evolved as natural inhibitors of G6PD further strengthens the connection between the primate adrenal androgen system and tumor suppression (Jiang *et al.* 2011, Hong *et al.* 2014). In a lineage-exclusive manner, then, these evolutionary innovations enabled the kill switch mechanism to be deployed in somatic cells of Anthropoid primates with great efficiency, preventing neoplasia from becoming a significant cause of death in this lineage during periods of their life span characterized by high levels of circulating DHEAS. The fine tuning of kill switch function, mediated by duration and peak levels of adrenal secretion of DHEAS, then evolved in a species-specific manner as Anthropoid primates deployed different strategies to exploit their different environmental niches. In humans, such strategies included the harnessing of fire, resulting in species-specific exposure to polycyclic aromatic hydrocarbons (PAH) and other carcinogens produced by the incomplete combustion of organic materials. Such continuous high-level carcinogen exposure in unventilated primitive habitats may have exerted a selective pressure favoring humans with higher peak circulating levels of DHEAS, and therefore, an optimized kill switch tumor suppression mechanism (Supplementary Section 1, see section on supplementary data given at the end of this article). We note that other species-specific genomic alterations related to PAH exposure have already been reported (Gassmann *et al.* 2010, Hubbard *et al.* 2016). We further note that PAHs are particularly potent inactivators of p53 function, not only through mutation (Tretyakova *et al.* 2002), but also by inducing an array of p53-inhibiting microRNAs (Gordon *et al.* 2005); they are therefore likely to be exceptional activators of the adrenal androgen-mediated kill switch.

### The human adrenal androgen-mediated kill switch remains set for prehistoric *Homo* species

**Figure 4:**
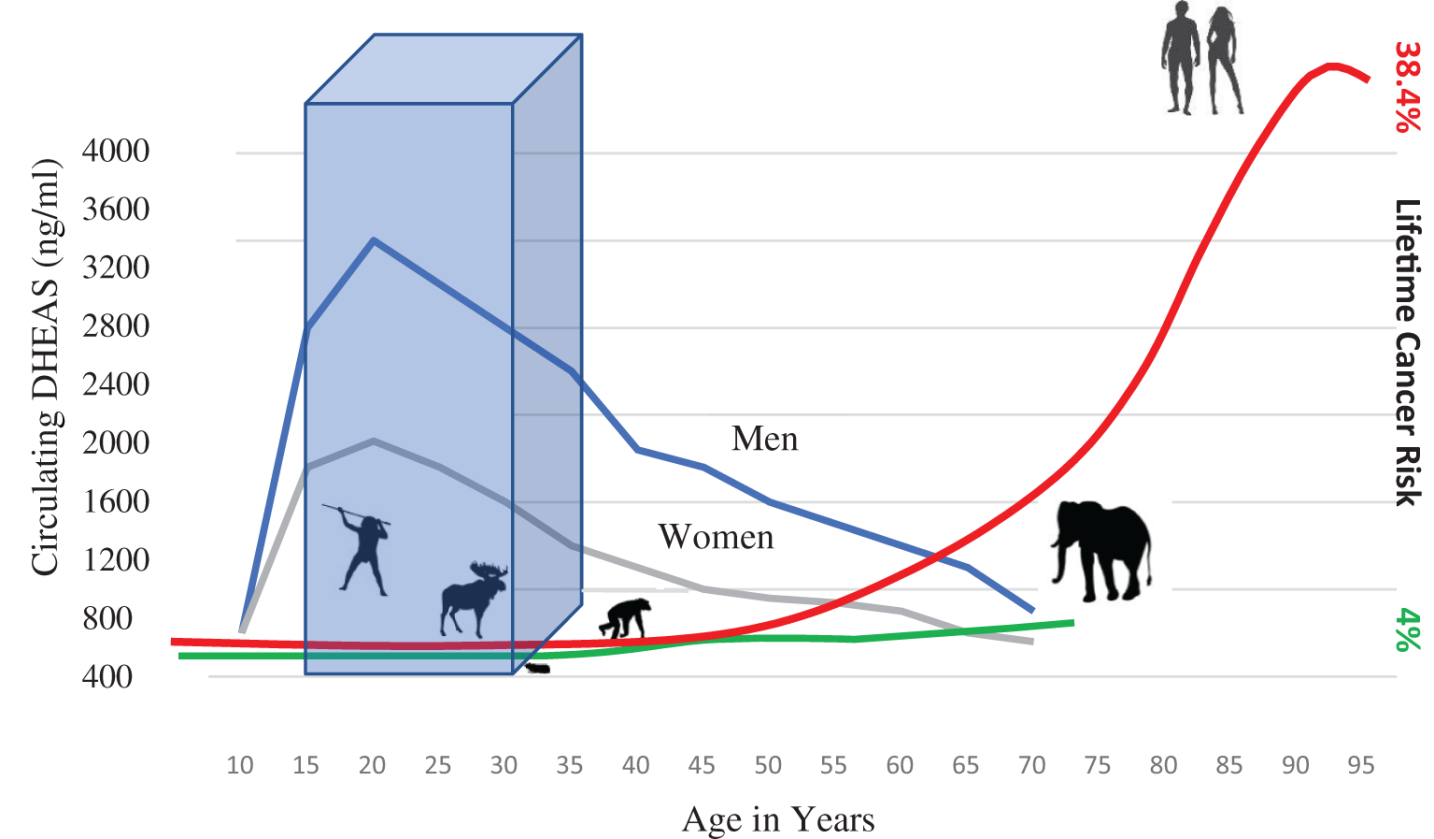
Species-specific kill switch tumor suppression systems targeting G6PD. In humans, circulating DHEAS (blue and grey lines), and therefore, kill switch function is maintained at optimal levels only up until about age 25 years – the life expectancy for humans for most of our existence as a species. The adrenal androgen-mediated kill switch evolved to provide protection for such short human life spans (blue rectangular prism), and declines sharply thereafter. Because modern humans live for much longer periods of time, the phenomenon of exponentially increasing cancer risk with increasing age is observed (red line). Species such as the elephant, moose and naked mole rat, which use tumor suppression systems that do not decline with age, experience little or no increased risk of cancer as they age (green line). Cancer risk redrawn after Cancer Incidence by Age (http://www.cancerresearchuk.org/health-professional/cancer-statistics/incidence/age). Lifetime risk of 38.4% (https://www.cancer.gov/about-cancer/understanding/statistics). Four percent cancer risk of most long-lived mammals from Abegglen *et al.* (2015). Circulating DHEAS levels redrawn after (dePeretti & Forest 1976, Parker & Odell 1980, Vermuelen 1980, Orentreich *et al.* 1984, Labrie *et al.* 1997).

As noted earlier, modern humans have one of the highest life-time risks of developing malignant cancer in the entire animal kingdom. At 38.4%, this risk is more than an order of magnitude higher than that of most other long-lived species, for example, the elephant. As is evident in Fig. 4, modern humans experience an age-associated exponential increase in cancer risk as their circulating levels of DHEAS decline. However, did this relationship hold for primitive *Homo sapiens*? Humans have experienced a recent extreme increase in longevity. For the vast majority of our species’ existence, life was short. Weiss and others have calculated a probable life expectancy at birth for primitive man of about 25 years; survivability to adulthood of about 50%; 88% mortality before the age of 30 years and generation times of about 20–25 years (Weis 1981, 1984, Kennedy 2003, Trinkaus 2011). World Health Organization data shows that as recently as 1900, global life expectancy at birth in the undeveloped world was just 26.5 years (Roser 2018), and even today ranges from 21 to 37 years for different extant hunter-gatherer tribes that have limited access to modern healthcare (Gurven & Kaplam 2007). Only in the last 50–75 years have dramatic improvements in public health enabled the majority of humans, at least in industrialized countries, to live into old age (Olshansky *et al.* 1990, Smith 1993). The life-time risk of developing a malignant tumor during virtually all of our species’ prehistoric existence was thus almost certainly in the 4% range of other long-lived mammals. However, the adrenal androgen-mediated kill switch, which evolved to protect during a human life span that generally did not exceed 25 years, did not keep pace with the increasing longevity of modern humans (Fig. 4). The excursion into old age that is being made by modern humans is thus being conducted without the protection of the natural adrenal androgen-mediated kill switch, which is still set to protect only for the very short life span of our ancestors.

### Species-specific tumor suppression in the African elephant and the naked mole rat

Other long-lived mammals, lacking the circulating DHEAS and the G6P accumulation-enabling G6PC promoter tetrad of the Anthropoid primates, have developed alternative species-specific tumor suppression systems. A recent study showed that the lifetime risk for an elephant dying of cancer is less than 5%, with no apparent increase in cancer risk with increasing age (Abegglen *et al.* 2015). The explanation for this low and constant cancer rate appears to be found in the elephant genome. In addition to the two alleles of the *p53* tumor suppressor found in humans, the elephant genome has an additional nineteen *p53* retro-pseudogenes (*p53p*), many of which appear to be translated into protein (Abegglen *et al.* 2015, Sulak *et al.* 2016). In a comparison of elephant cells (two active *p53* alleles and 19 *p53p*), normal human cells (two active *p53* alleles), and cells from patients with Li Fraumeni syndrome (one active *p53* allele), Abegglen *et al.* (2015) observed that the induction of apoptosis in response to DNA damage in elephant cells (14.64%; 95% CI, 10.91–18.37%; *P* < 0.001) was twofold higher than in normal human cells (7.17%; 95% CI, 5.91–8.44%; *P* < 0.001) and five-fold higher than in cells from patients with Li Fraumeni syndrome (2.71%; 95% CI, 1.93–3.48%). In parallel studies in which siRNA were used to block translation of *p53p*, the increased apoptotic rate in elephant cells exposed to DNA damaging agents was significantly reduced (Sulak *et al.* 2016). These data demonstrate that *p53p* pseudogenes contribute to the sensitivity to induction of apoptosis, although the exact mechanism of action remains to be clarified.

The naked mole rat (NMR) is a mouse-sized creature that leads an entirely subterranean existence, has the longest life span of any rodent at 28–35 years, and is also unique in its virtually complete resistance to cancer in its natural habitat (Gorbunova *et al.* 2014, Lagunas-Rangel & Chavez-Valenica 2017). It has recently been discovered (Tang *et al.* 2016) that the NMR has 17 retropseudogenes corresponding to the phosphatase and tensin homologue (*PTEN*) tumor suppressor. *PTEN* and *p53* tumor suppressors are often given co-status as ‘the guardians of the genome’, because both are transcription factors that activate complex programs of apoptosis in cells that suffer potentially tumorigenic levels of DNA damage (Yin & Shen 2008, Ryan 2011). What has gone essentially unnoticed is that, like DHEA, both p53 and PTEN are direct inhibitors of G6PD (Jiang *et al.* 2011, Hong *et al.* 2014). Thus, the possibility exists that *p53p* in the elephant, and the *PTEN* retropseudogenes (*PTENp*) in the NMR, constitute effectors of species-specific kill switch mechanisms that parallel the adrenal androgen-mediated kill switch of primates by targeting G6PD for lethal inhibition. The naked mole rat has also been discovered to have a species-specific high molecular weight hyaluronic acid isoform (Tian *et al.* 2013) which, by virtue of its anti-oxidant function, could be part of such a G6PD-targeting kill switch system (Supplementary Section 3).

### Primordial role of DHEAS in kill switch maintenance of germline genomic integrity?

Although circulating DHEAS and the autosomal kill switch mechanism that utilizes it are species-specific phenomena, the uncompetitive inhibition kinetics of DHEA with respect to G6PD are not; they exist in all animal species with endogenous DHEA. This suggests that such uncompetitive, potentially irreversible inhibition kinetics must have been selected for in all species with DHEA, not just those limited few with circulating DHEAS. What then, was the overall selective pressure for such an unusual form of enzyme inhibition, targeting such a critical enzyme as G6PD? The maintenance of germ cell genomic integrity is a critical task that must be conducted with extremely high fidelity, particularly with respect to oocytes of mammals, since female mammals make a comparatively enormous investment in their offspring. Considering the finite number of oocytes that female mammals carry, this large investment in their offspring is best initiated using the highest quality oocytes available. This is most efficiently accomplished by eliminating oocytes from the reproductive pool that have experienced decrement in their genomic integrity. Such genomic integrity must be maintained for very long periods of time – decades in some species, including humans – as oocytes are arrested in prophase of meiosis I between homologous chromosome recombination and ovulation. It has been established in lower animals such as *Caenorhabditis elegans* and *Nematostella vectensis* that the primordial role of the p53 family was to maintain the genomic integrity of germ cells (Lettre *et al.* 2004, Pankow & Bamberger 2007). TP53 family members p63 and p73 have strong structural similarity to p53 and are known to bind as a transcription factor to many of the same gene targets as p53 (Strano *et al.* 2001). Whereas p53 is considered to be the guardian of the somatic cell genome, Tap63 (the full-length version of p63) is considered to be the guardian of the germ line, and oocytes and spermatozoa with damaged genomes are extinguished by its action (Suh *et al.* 2006, Beyer *et al.* 2011, Napoli & Flores 2016, Gebel *et al.* 2017). It has been elegantly demonstrated that Tap63alpha remains kinetically trapped in an inactive dimer form, a sort of spring-loaded mechanism that can respond instantly to oocyte DNA damage. Upon detection of such oocyte DNA damage, Tap63alpha undergoes rapid phosphorylation-induced tetramerization and activation (Coutandin *et al.* 2016), resulting in a rapid upregulation of NFKB activity and the induction of apoptosis (Sen *et al.* 2011). We propose that DHEAS participates in that role with Tap63alpha and that this cooperation in guarding germline DNA is the selective force behind the evolution of the uncompetitive inhibition kinetics of DHEA with respect to G6PD. Whereas the kill switch triggered by p53 inactivation in human somatic cells requires circulating DHEAS, and therefore, cannot operate in species lacking circulating DHEAS, this is not so for germ cells of animals with ovaries and testes because these are among the limited organs in which DHEAS is synthesized locally. Oocytes have in fact been found to have unexpectedly large standing pools of DHEAS (Dehennin *et al.* 1987, Jimena *et al.* 1992, Haccard *et al.* 2012). This suggests that DNA-damage-induced tetramerization of Tap63alpha triggers NFKB in oocytes, which then simultaneously activates steroid sulfatase to produce DHEA from this large standing pool of oocyte DHEAS. In oocytes, NFKB will also inactivate G6PC to permit the accumulation of G6P (Grempler 2004). By satisfying the requirements for irreversible uncompetitive inhibition of G6PD, i.e., high concentrations of inhibitor and substrate, the kill switch mechanism targeting G6PD with irreversible uncompetitive inhibition can thus be unleashed as necessary in the germ cell compartment of all animals that can produce gonadal DHEAS. This appears to also be true for spermatogonia, where a protective role has already been observed for DHEAS (Papadopoulos *et al.* 2017) and for p63 (Beyer *et al.* 2011).

Also of interest is the observation that all placental mammals have a well-developed adrenal fetal zone producing extraordinarily high levels of DHEAS throughout gestation, with such levels sharply falling off to near zero at birth (Pashen *et al.* 1982, Conley *et al.* 1994, Raeside *et al.* 1997, Parker 1999 Rainey *et al.* 2004). The extraordinary rates of DNA replication occurring during fetal development should make the fetus extremely sensitive to neoplastic transformation; yet, such transformation is rare. The possibility therefore exists that, among species employing fetal adrenal synthesis of DHEAS, its uncompetitive inhibition kinetics with respect to G6PD may have been under selective pressure to maintain not only germline integrity, but also fetal somatic cell integrity. Primates then duplicated elements of this system to protect their somatic cells into adulthood, evolving adrenal *zona reticularis* with the ability to synthesize and secrete DHEAS into the circulation following adrenarche, and the G6PC promoter motif that enables accumulation of G6P substrate.

### Is the human-specific, aging-associated exponential increase in cancer risk unalterable?

**Figure 5:**
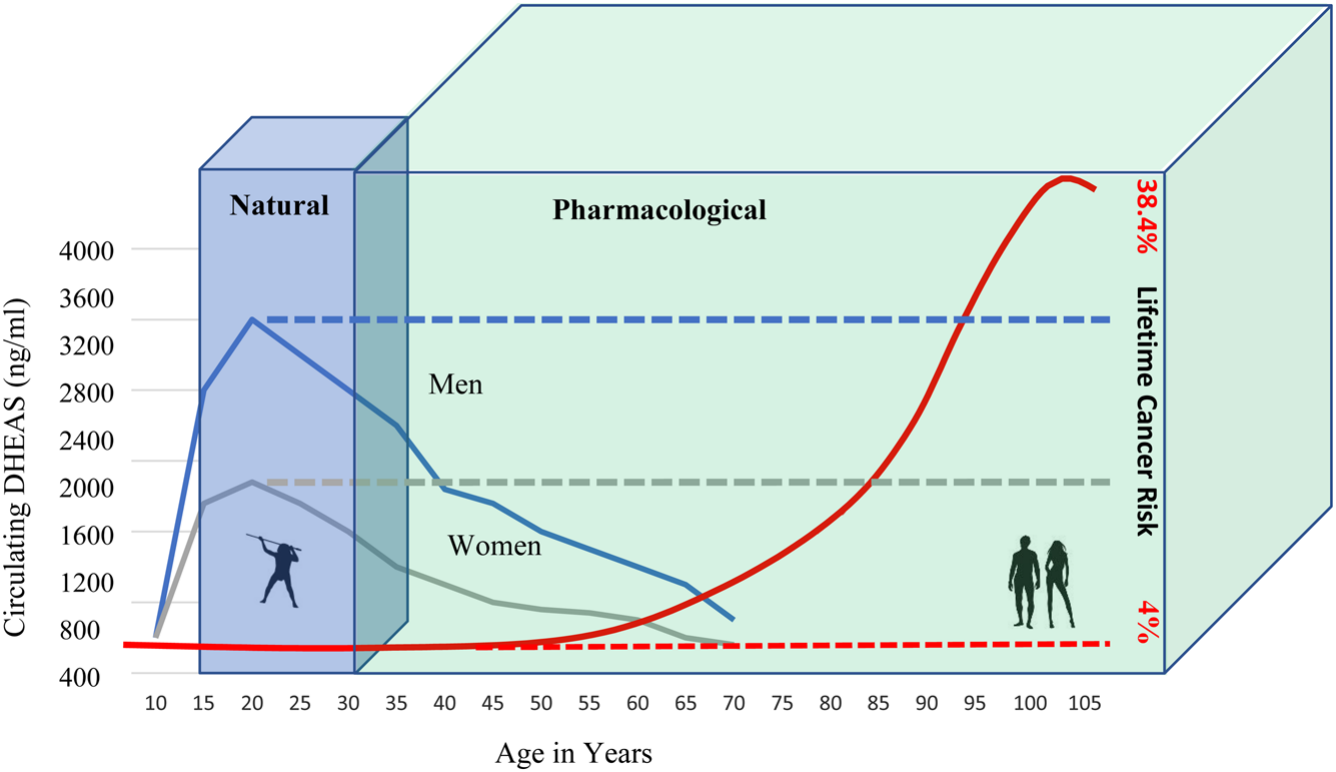
Potential for pharmacologic extension of adrenal androgen-mediated kill switch. Humans are protected by their natural adrenal androgen-mediated kill switch only until about age 30 (blue rectangular prism). Circulating DHEAS levels decline dramatically thereafter in both men and women (solid blue and grey lines, respectively), resulting in a species-specific exponential increase in cancer risk with increasing age (solid red line). However, optimum DHEAS levels can be pharmacologically maintained (dashed blue and grey lines) throughout life into old age (green rectangular prism). If the analogy holds with other long-lived species such as chimpanzees and elephants, in which kill switch mechanisms targeting G6PD are maintained throughout life, pharmacological maintenance of peak DHEAS levels throughout the modern human life span may normalize the age-associated increase in human cancer risk to that of most other species (dashed red line).

A perhaps surprising revelation supported by recent data is that the exponential increase in cancer risk with increasing age appears to be a human-specific phenomenon that does not occur in most other species, in which cancer risk with increasing age shows a relatively flat trajectory. The extremely low and flat cancer risk experienced by elephants and many other species throughout their entire lifetimes (Abegglen *et al.* 2015) thus appears to represent the evolutionary norm, making the exponential increase in cancer risk with increasing age observed in humans the outlier. Unlike elephants, or naked mole rats, which are long-lived species whose mechanisms of tumor suppression are genetic and effected by constitutive macromolecules, the adrenal androgen-mediated kill switch of humans is effected by a small molecule, DHEAS, and is therefore potentially subject to pharmacological manipulation. This raises the possibilities that (a) the failure of kill switch evolution to keep pace with modern human life expectancy might be overcome by pharmacologically maintaining circulating DHEAS at its peak level throughout the modern human life span; and (b) extrapolating from other long-lived animals who appear to maintain parallel kill switch mechanisms over their lifetimes, the human-specific phenomenon of exponentially increasing cancer risk with increasing age might be eliminated by such pharmacological maintenance of the adrenal androgen-mediated kill switch. It will therefore be important to determine if such pharmacological reconstitution of the kill switch mechanism throughout the lifespan can normalize the 38.4% lifetime cancer risk of modern humans to the low, flat cancer risk experienced by virtually all other long-lived mammals (Fig. 5).

### Diet and the kill switch

Factors in addition to the adrenal androgen-mediated kill switch clearly also modulate cancer risk in aging primates. Thus, circopithecidaen primates such as Rhesus monkeys enjoy very low cancer risk throughout life (Lombard & Witte 1959), despite a gradual age-related decline in plasma DHEAS (Kemnitz *et al.* 2000, Sorwell & Urbanski 2013). Unlike modern humans, however, primates in captivity are subjected to rigorously controlled diets that maintain them at optimum weights and percentage body fat. Since caloric restriction is well known to inhibit carcinogenesis (Brandhorst & Longo 2016, Kopeina *et al.* 2017), and caloric excess to promote it (Allott & Hursting 2015, Hopkins *et al.* 2016), the controlled diets of primates in captivity may account for some portion of their reduced lifetime risk of cancer. However, Rhesus monkeys also have only one-tenth the body mass of adult humans, so their smaller body size also contributes to species-specific tumor suppression, as we have discussed for the mouse. Body size can also modulate *human* cancer risk. Thus, the recent ‘million women study ‘, which followed 1,297,124 women for a median time of 9.4 years each, reported an overall 16% increase in cancer risk for every 10 cm (4 inches) in height above average (Green *et al.* 2011). This association of increased cancer risk with increased height has been confirmed by additional studies performed in 144,701 women (median follow-up, 12 years) (Kabat *et al.* 2013), and in 310,000 male and female UK Biobank participants (Ong *et al.* 2018). At the opposite end of the spectrum, studies of dwarf humans with Laron Syndrome – one of which studies lasted 57 years – demonstrated a near total absence of cancer in these long-lived, small bodied humans (Janecka *et al.* 2016, Laron & Kauli 2016). Extrapolating this finding, the evolutionary trajectory of humans through spacetime may have taken advantage of small body size during childhood as an inexpensive means of tumor suppression, such that combined with the canonical p53 repertoire, it proved sufficient to minimize cancer risk during this developmental phase. It is only in preparation for the increased stature occurring with puberty that the adrenal androgen-mediated kill switch tumor suppressor system becomes necessary, and the *zona reticularis* undergoes its extraordinary activation and release of DHEAS into the circulation. Increased adult body size would clearly have been adaptive for survival in the primitive landscape in which predation by large carnivores, and intertribal warfare, constituted major selective pressures. However, such increased stature would have come at the price of increased cancer risk if only the canonical p53 repertoire was operative. Thus, as primitive humans progressed into adulthood, high levels of circulating DHEAS required for kill switch tumor suppression may have enabled them to maintain the low cancer risk of their juvenile phase while accumulating the increased body mass that enhanced their probability of survival. This line of reasoning suggests that the loss of functional levels of circulating DHEAS as modern humans surpass the primitive life span may be responsible for the excess cancer risk associated with increased height and that pharmacological reconstitution of DHEAS levels might eliminate such excess risk.

As previously noted, humans have by far the highest peak levels of circulating DHEAS, followed by the chimpanzee (Blevins *et al.* 2013). Chimpanzees weigh 40–60 kg (88–130 pounds), about the same as primitive humans (McHenry 1976). With respect to diet, it is important to note that humans and chimpanzees are the only two primates that regularly eat meat and that the consumption of red meat is known to be carcinogenic (Johnson 2017). Chimpanzees consume only a fraction of the meat that humans do, and neither did they harness fire, as humans did. Their species-specific trajectory through spacetime thus did not involve exposure to the heterocyclic amines, N-nitrosamines and polycyclic aromatic hydrocarbons that are formed in the processing of meat by heat (Gu *et al.* 2011, Bellamri *et al.* 2018). Consumption of such heat-processed meat is well documented to be carcinogenic (Milton 1999, IARC 2015, Chiang & Quek 2017). The combination of less dependence upon dietary meat and a complete absence of exposure to the carcinogens in cooked meat clearly delineates the evolutionary trajectory of the chimpanzee as compared to humans. Chimpanzees therefore may have had a reduced requirement for the primate-specific adrenal androgen-mediated kill switch tumor suppression system, and this may account for the fact that their peak circulating levels of DHEAS are only about half what they are in humans (Blevins *et al.* 2013). In this regard, also consider other Anthropoid primates. Gorillas can weigh 140–180 kg (300–400 pounds) and orangutans 115 kg (250 pounds). However, both are vegetarian in their diets, and have little or no exposure to the carcinogens found in cooked meat. Their circulating DHEAS levels are, respectively, one-third and one-sixth that of chimpanzees, who do eat meat, albeit raw meat; and one-sixth and one-eleventh, respectively, that of humans who consume heat-processed meat (Bernstein *et al.* 2012). These facts suggest that the Anthropoid primate-specific adrenal androgen-mediated tumor suppressor system enabled the harnessing of fire by primitive humans, and the consequent exposure to the carcinogens produced in heat-processed meat selected for humans with the highest circulating levels of DHEAS, and hence, optimum function of the kill switch tumor suppressor system. Modern humans, however, consume far more heat-processed meat than our primitive ancestors had the opportunity to do, significantly increasing our exposure to the carcinogens created in such heat-processed meat – and also leading to the current epidemic of obesity. With respect to obesity, the U.S. Center for Disease Control (CDC) has recently reported that the average American woman today weighs as much as the average American man did in the 1960s, while the body mass of the average modern American male is now nearly double that estimated for primitive members of our species (McHenry 1976, Ogden & Carroll 2010). Such dramatic increases in body mass have put modern humans outside the limits under which the kill switch tumor suppression system evolved. Another negative consequence of obesity in modern humans is that fat expresses tissue-specific isoforms of steroid sulfatase, such that excessive accumulation of fat sequesters circulating DHEAS in this tissue (Dalla Valle *et al.* 2006), further degrading kill switch function in aging individuals who are overweight. Thus, the modern human diet focused as it is on the consumption of heat-processed meat, and the obesity commonplace in modern humans, place further significant constraints upon kill switch function in aging humans who have declining levels of circulating DHEAS.

### Exogenous DHEA

The age-associated loss of circulating DHEAS has prompted many hypotheses regarding the biological role of DHEA in humans. However, none of these hypotheses have been informed by knowledge of the kill switch mechanism. In humans, circulating levels of DHEA are kept safely in the low nanomolar range, several orders of magnitude below its IC_50_ for G6PD inhibition, while DHEAS circulates at micromolar levels that are just slightly under the IC_50_ for DHEA inhibition of G6PD (Gordon *et al.* 1986, Labrie *et al.* 1997). These natural conditions clearly evolved to prevent destruction of normal cells and tissues by irreversible catastrophic uncompetitive inhibition of G6PD, such that DHEAS will only be converted to DHEA in cells in which p53 inactivation has occurred or that require intracrine steroid hormone synthesis (Labrie 2015). Systemically administered DHEA is therefore likely to produce toxicities in humans that would not be observed in murine species and render the kill switch tumor suppression system inoperable via the induction of tolerance. There is also the potential for serious drug interactions, e.g., with carboxylic NSAIDs such as ketoprofen, the CoA conjugates of which have been shown to bind irreversibly to and inhibit G6PD (Asensio *et al.* 2006). For safety reasons, therapeutic administration of DHEA should therefore be limited to local administration, as for example in a recent treatment for vaginal atrophy approved by the U.S. Food and Drug Administration (Bouchard *et al.* 2016, Labrie *et al.* 2016). Most developed countries regulate DHEA as the potentially toxic compound that it is. Where DHEA is sold as a food supplement, there is no adequate mechanism available for adverse event reporting as there would be for drugs administered under a physician’s supervision.

## Conclusion: trouble in paradigm

The p53 gene was discovered almost 40 years ago (Kress *et al.* 1979, Lane & Crawford 1979, Linzer & Levine 1979), and its role as a major tumor suppressor was identified a decade later (Baker *et al.* 1989, Takahashi *et al.* 1989). The p53-knockout mouse model of human cancer has been a staple of cancer research for some 26 years (Donehower *et al.* 1992). The depth of infiltration of this model into the fabric of human cancer research is demonstrated by the fact that it has been accepted by the FDA as a preclinical model for human drug development for more than 20 years (FDA 1997). The use of this model system over this long period of time has created a paradigm in which mutations in p53 are considered to be linear initiators of carcinogenesis with virtually universal application independent of species, such that results in one species, e.g., the mouse, are thought to accurately translate to another, e.g., the human. Yet, new research in non-murine species (dog, elephant, naked mole rat, etc.) suggests that while p53 may be a universal *sensor* of mutagenic insult, many animals, including humans, adopt species-specific solutions to such insult and those species-specific solutions triggered by p53 inactivation appear frequently to converge mechanistically upon lethal inhibition of G6PD. These observations suggest that the focus of nature’s anti-cancer effort is the singularity. In this way, nature suppresses cancer at its most vulnerable point, at the level of the initial, potentially transformed cell, before it has initiated the explosion of diversification that has made clinical cancer incurable up to now. This appears to be how the elephant suppresses cancer throughout its long life, with its species-specific method to enhance its p53-mediated kill switch system. It also appears to be how chimpanzees and other great apes suppress cancer (McClure 1973, Hill *et al.* 2001, Brown *et al.* 2009), capitalizing upon primate-specific circulating DHEAS and *G6PC* promoter motifs, as well as the uncompetitive G6PD inhibition kinetics of DHEA. We believe that the species-specific DHEAS-mediated kill switch is fundamental to cancer suppression in humans.

Cancer continues to be among the leading causes of death worldwide and is predicted to soon overtake cardiovascular disease as the number one cause of death in Western countries (Ma *et al.* 2015). This trend will accelerate as progress in the treatment of cardiovascular disease, influenza and other major killers outstrip progress in the treatment of cancer, and more people who would have died of these other diseases survive into old age. In 2012, there were 14 million new cancer cases and 8.2 million cancer deaths worldwide (WHO 2018). The number of new cancer cases is expected to increase to 23.6 million annually by 2030 (https://www.cancer.gov/about-cancer/understanding/statistics; http://www.who.int/mediacentre/factsheets/fs297/en/), which will place an incredible burden on healthcare delivery. In China, cancer is already the number one cause of death, with 4.3 million newly diagnosed cases of invasive cancer annually and 2.3 million cancer deaths (Chen *et al.* 2015). Clearly, with the increases in longevity being experienced by our species, cancer has already reached epidemic proportions. As we have not yet reached what is believed to be the maximum human lifespan (Gavrilov *et al.* 2017), the current cancer epidemic will only become magnified in the future, far outstripping the ability of all currently applied strategies to stem it.

**Figure 6:**
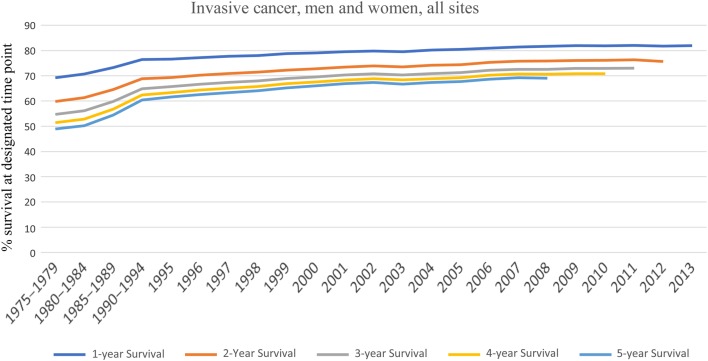
Treatment outcome in invasive cancer appears to be approaching an asymptotic boundary. Data from NCI SEER Cancer Statistics Review (CSR) 1975–2014. Updated June 28, 2017 (https://surveillance.cancer.gov/statistics/types/survival.html).

Critical review reveals that over the past 40 years, improvements in patient survival for most types of cancer have been nominal. While cancer death rates have declined each year since 2000 in developed countries, most of this reduction is thought to be due to the decreased use of tobacco (Malvezzi *et al.* 2017). Even the most highly touted new treatment modalities extend life only marginally and are rapidly overcome by the resistance made possible by tumor heterogeneity (Delyon *et al.* 2015, Iafolla & Juergens 2017). Exceptions to this rule exist, but they are rare (McDermott *et al.* 2014, Delyon *et al*. 2015, Callahan *et al.* 2017), and are unlikely to contribute in a significant way to overall survival. Data from the U.S. National Cancer Institute (https://www.cancer.gov/about-cancer/understanding/statistics) demonstrate that 2-year survival for invasive cancers has improved less than 7% over the past 27 years and appears to be at an asymptotic boundary beyond which further improvement may be negligible (Fig. 6).

Furthermore, a not insignificant fraction of the improvement in survival that has been achieved may be due to advances in the supportive care that cancer patients now receive, rather than on primary medical intervention itself (McCorkle *et al.* 2000, Irwin *et al.* 2013). In one study evaluating supportive care of cancer patients (nutrition, psychological intervention, etc.), 2-year survival among late-stage cancer patients receiving such care was 67%, compared to 40% among control cases receiving only medical intervention. When Cox’s proportional hazard model was used to adjust for baseline covariates, the relative hazard of death in the control group was 2.04 (CI: 1.33–3.12; *P* = 0.001); i.e., patients with invasive cancers who received only medical intervention were twice as likely to die during this period of time (McCorkle *et al.* 2000).

Such data do not encourage a ‘stay the course’ approach to cancer research, but rather suggest that something is fundamentally wrong with the paradigms that have been guiding this endeavor for at least three decades. The unanticipated existence of an essentially human-specific adrenal androgen-mediated kill switch tumor suppression mechanism clearly undermines much of the research that has been performed *ex vivo*, *in vivo* and *in vitro* over this long period of time:

- *ex vivo* analysis of p53 mutations in human tumors may have done little more than reveal evidence of a kill switch tumor suppressor system malfunction caused by an artifact of hominid evolution – circulating DHEAS that declines sharply once the primitive human life span, for which it evolved, is exceeded;
- the fundamental differences in tumor suppression mechanisms between human and murine species appear to completely disqualify the latter as *in vivo* models of human cancer; and
- *in vitro* studies utilizing human cells in which culture conditions do not model *in vivo* circulating DHEAS have de-evolved human cells to the equivalent of non-informative murine cells.

To the extent that these criticisms are valid, fundamental flaws in our operating paradigms have been leading us far off course for decades. Without appropriate course corrections, we may continue to generate species-specific cancer data for species other than our own.

The slow, very meager progress in prolonging cancer survival, the fact that such survival appears to be at an asymptotic boundary beyond which any further progress may be impossible, and the extreme, accelerating and clearly unsustainable costs of new cancer drugs that only minimally extend life (Sidduqui & Rajkumar 2012, Cohen 2017, Davis *et al.* 2017, Jackson & Nahata 2017, Prasad & Mailankody 2017, Carrera *et al.* 2018, Dranitsaris *et al.* 2018), all indicate the necessity for reappraisal of the current paradigm in which developed tumors are the target for virtually all of our anti-cancer research efforts. It may be time to redirect our labors and research expenditures toward understanding the singularity, the apparent focus of nature’s major effort at tumor suppression. If tumor complexity has been the Gordian knot of the cancer problem, preventing real progress in cancer treatment, then reactivating a kill switch made latent by an age-related decline in DHEAS may represent Alexander’s blade. The adrenal androgen-mediated kill switch tumor suppression system has the singularity as its target, and its evolutionary programming for a prehistoric, not a modern life span, may be responsible for the anomaly of an exponentially increasing rate of cancer with increasing age in our species. Singularities occurring in aging modern humans experience a diminishing capacity to undergo irreversible G6PD inhibition because of the dramatically declining levels of circulating DHEAS and consequent inability to trigger the kill switch mechanism. While this was not problematic for our ancestors who rarely reached the age of 30 years, it is problematic for modern humans who regularly live into and beyond their ninth decade. The life-long low, flat cancer risk observed in other long-lived animals that employ parallel, but life-long species-specific tumor suppression strategies, suggests that a similarly life-long low, flat cancer risk may be achievable in humans; that is, there appears to be no *a priori* reason, such as accumulated genomic damage, that makes an age-related increase in human cancer unavoidable. Rather, an approximately 4% lifetime cancer risk may be the norm for all species, including *Homo sapiens*. The lesson from other long-lived species appears to be that kill switch mechanisms that function throughout life extinguish almost all potentially tumorigenic damage at the level of the singularity. If pharmacological maintenance of DHEAS at peak levels establishes life-long functionality of the adrenal androgen-mediated kill switch, humans might join the majority of the animal kingdom in which death from cancer is a rare event and has little to do with advancing age. Determining what the true background risk of cancer is in the presence of such a fully functional, life-long adrenal androgen-mediated kill switch tumor suppression system should therefore be a primary goal of our species.

## Supplementary Material

Supplementary Section 1

Supplementary Section 2

Supplementary Section 3

## Declaration of interest

Dr Nyce is a listed inventor on patent applications related to methods to maintain function of the kill switch tumor suppression system.

## Funding

This work was funded in part by the U.S. Food and Drug Administration (G003126-C-2195-SR1891; G003126-C-2195-SR1892; G003126-C-2195-SR1893; G003126-C-2195-SR1894; G003126-C-2195-SR1895 to J W N).

## References

[bib1] Abbadi S Rodarte JJ Abutaleb A Lavell E Smith CL William Ruff W Jennifer Schiller J Alessandro Olivi A Andre Levchenko A Hugo Guerrero-Cazares H, ***et al*** 2014 Glucose-6-phosphatase is a key metabolic regulator of glioblastoma invasion. Molecular Cancer Research 12 1547–1559. (10.1158/1541-7786.MCR-14-0106-T) 25001192 PMC4233174

[bib2] Abegglen LM Caulin AF Chan A Lee K Robinson R Campbell MS Kiso WK Schmitt DL Waddell PJ Bhaskara S, ***et al*** 2015 Potential mechanisms for cancer resistance in elephants and comparative cellular response to DNA damage in humans. JAMA 314 1850–1860. (10.1001/jama.2015.13134) 26447779 PMC4858328

[bib3] Ahmad AS Ormiston-Smith N Sasieni PD 2015 Trends in the lifetime risk of developing cancer in Great Britain: comparison of risk for those born from 1930 to 1960. British Journal of Cancer 112 943–947. (10.1038/bjc.2014.606) 25647015 PMC4453943

[bib4] Alexandersen P Haarbo J Byrjalsen I Lawaetz H Christiansen C 1999 Natural androgens inhibit male atherosclerosis: a study in castrated, cholesterol-fed rabbits. Circulation Research 84 813–819. (10.1161/01.RES.84.7.813) 10205149

[bib5] Allott EH Hursting SD 2015 Obesity and cancer: mechanistic insights from transdisciplinary studies. Endocrine-Related Cancer 22 R365–R386. (10.1530/ERC-15-0400) 26373570 PMC4631382

[bib6] Asensio C Levoin N Guillaume C Guerquin MJ Rouguieg K Chretien F Chapleur Y Netter P Minn A Lapicque F 2006 Irreversible inhibition of glucose-6-phosphate dehydrogenase by the coenzyme A conjugate of ketoprofen: a key to oxidative stress induced by non-steroidal anti-inflammatory drugs? Biochemical Pharmacology 73 405–416. (10.1016/j.bcp.2006.09.026) 17094951

[bib7] Ashley PF Frank LA Schmeitzel LP Bailey EM Oliver JW 1988 Effect of oral melatonin administration on sex hormone, prolactin, and thyroid hormone concentrations in adult dogs. Journal American Veterinary Medical Association 215 1111–1115. 10530323

[bib8] Avivi A Ashur-Fabian O Joel A Trakhtenbrot L Adamsky K Goldstein I Amariglio N Rechavi G Nevo E 2007 P53 in blind subterranean mole rats – loss-of-function versus gain-of-function activities on newly cloned Spalax target genes. Oncogene 26 2507–2512. (10.1038/sj.onc.1210045) 17043642

[bib9] Axelson M Graham CE Sjövall J 1984 Identification and quantitation of steroids in sulfate fractions from plasma of pregnant chimpanzee, orangutan, and rhesus monkey. Endocrinology 114 337–344. (10.1210/endo-114-2-337) 6690281

[bib10] Baker SJ Fearon ER Nigro JM Hamilton SR Preisinger AC Jessup JM vanTuinen P Ledbetter DH Barker DF Nakamura Y, 1989 Chromosome 17 deletions and p53 gene mutations in colorectal carcinomas. Science 244 217–221. (10.1126/science.2649981) 2649981

[bib11] Behringer V Hohmann G Stevens JM Weltring A Deschner T 2012 Adrenarche in bonobos (*Pan paniscus*): evidence from ontogenetic changes in urinary dehydroepiandrosterone-sulfate levels. Journal of Endocrinology 214 55–65. (10.1530/JOE-12-0103) 22562655

[bib12] Bélanger B Bélanger A Labrie F Dupont A Cusan L Monfette G 1989 Comparison of residual C-19 steroids in plasma and prostatic tissue of human, rat and guinea pig after castration: unique importance of extra-testicular androgens in men. Journal of Steroid Biochemistry 32 695–698. (10.1016/0022-4731(89)90514-1) 2525654

[bib13] Bellamri M Xiao S Murugan P Weight CJ Turesky RJ 2018 Metabolic activation of the cooked meat carcinogen 2-amino-1-methyl-6-phenylimidazo[4,5-b]pyridine in human prostate. Toxicology Science 163 543–556. (10.1093/toxsci/kfy060) PMC5974788 29596660

[bib14] Bernstein RM Sterner KN Wildman DE 2012 Adrenal androgen production in catarrhine primates and the evolution of adrenarche. American Journal of Physical Anthropology 147 389–400. (10.1002/ajpa.22001) 22271526 PMC4469270

[bib15] Beyer U Moll-Rocek J Moll UM Dobbelstein M 2011 Endogenous retrovirus drives hitherto unknown proapoptotic p63 isoforms in the male germ line of humans and great apes. PNAS 108 3624–3629. (10.1073/pnas.1016201108) 21300884 PMC3048127

[bib16] Bird IM 2012 In the zone: understanding zona reticularis function and its transformation by adrenarche. Journal of Endocrinology 214 109–111. (10.1530/JOE-12-0246) 22700191

[bib17] Bjørnerem A Straume B Oian P Berntsen GK 2006 Seasonal variation of estradiol, follicle stimulating hormone, and dehydroepiandrosterone sulfate in women and men. Journal of Clinical Endocrinology and Metabolism 91 3798–3802. (10.1210/jc.2006-0866) 16835279

[bib18] Blevins JK Coxworth JE Herndon JG Hawkes K 2013 Brief communication: adrenal androgens and aging: female chimpanzees (*Pan troglodytes*) compared with women. American Journal of Physical Anthropology 151 643–648. (10.1002/ajpa.22300) 23818143 PMC4412270

[bib19] Borst GHA Vroege C Poelma FG Zwart P Strik WJ Peters JC 1972 Pathological findings on animals in the Royal Zoological Gardens of the Rotterdam Zoo during the years 1963, 1964, and 1965. Acta Zoologica et Pathologica Antverpiensia Editum Consilio 56 3–20. 4203108

[bib20] Bouchard C Labrie F Derogatis L Girard G Ayotte N Gallagher J Cusan L Archer DF Portman D Lavoie L, ***et al*** 2016 Effect of intravaginal dehydroepiandrosterone (DHEA) on the female sexual function in postmenopausal women: ERC-230 open-label study. Hormones Molecular Biology and Clinical Investigation 25 181–190. (10.1515/hmbci-2015-0044) 26725467

[bib21] Brandhorst S Longo VD 2016 Fasting and caloric restriction in cancer prevention and treatment. Recent Results in Cancer Research 207 241–266. (10.1007/978-3-319-42118-6_12) 27557543 PMC7476366

[bib22] Brown SL Anderson DC Dick EJJ Guardado-Mendoza R Garcia AP Hubbard GB 2009 Neoplasia in the chimpanzee (*Pan* spp.). Journal of Medical Primatology 38 137–144.(10.1111/j.1600-0684.2008.00321.x) 19367738 PMC2893876

[bib23] Buffenstein R 2008 Negligible senescence in the longest living rodent, the naked mole-rat: insights from a successfully aging species. Journal of Comparative Physiology B 178 439–445.(10.1007/s00360-007-0237-5) 18180931

[bib24] Callahan MK Kluger H Postow MA Segal NH Lesokhin A 2017 Nivolumab plus ipilimumab in patients with advanced melanoma: updated survival, response, and safety data in a phase 1 dose-escalation study. Journal of Clinical Oncology 36 391–398. (10.1200/jco.2017.72.2850) 29040030 PMC5946731

[bib26] Carrera PM Kantarjian HM Blinder VS 2018 The financial burden and distress of patients with cancer: understanding and stepping up action on the financial toxicity of cancer treatment. CA: A Cancer Journal for Clinicians 68 153–165. (10.3322/caac.21443) 29338071 PMC6652174

[bib27] Castrogiovanni C Waterschoot B De Backer O Dumont P 2017 Serine 392 phosphorylation modulates p53 mitochondrial translocation and transcription-independent apoptosis. Cell Death and Differentiation 25 190–203. (10.1038/cdd.2017.143) 28937686 PMC5729520

[bib28] Chen W Zheng R Baade PD Zhang S Zeng H 2015 Cancer statistics in China, 2015. CA: A Cancer Journal for Clinicians 66 115–132. (10.3322/caac.21338) 26808342

[bib29] Chiang VS Quek SY 2017 The relationship of red meat with cancer: effects of thermal processing and related physiological mechanisms. Critical Reviews in Food Science Nutrition 57 1153–1173. (10.1080/10408398.2014.967833) 26075652

[bib30] Cimons M Getlin J Maugh TH 1998 Cancer drugs face long road from mice to men. Los Angeles Times, May 6, 1998. (available at: http://articles.latimes.com/1998/may/06/news/mn-46795)

[bib31] Cohen D 2017 Cancer drugs: high price, uncertain value. BMJ 359 j4543 (10.1136/bmj.j4543) 28978508 PMC5695518

[bib32] Conley AJ Rainey WE Mason JI 1994 Ontogeny of steroidogenic enzyme expression in the porcine conceptus. Journal of Molecular Endocrinology 12 155–165. (10.1677/jme.0.0120155) 8060480

[bib33] Contente A Zischler H Einspanier A Dobbelstein M 2003 A promoter that acquired p53 responsiveness during primate evolution. Cancer Research 63 1756–1758. 12702557

[bib34] Cooks T Pateras IS Tarcic O Solomon H Schetter AJ 2013 Mutant p53 prolongs NF-KB activation and promotes chronic inflammation and inflammation-associated colorectal cancer. Cancer Cell 23 634–646. (10.1016/j.ccr.2013.03.022) 23680148 PMC3657134

[bib35] Cornish-Bowden A 1986 Why is uncompetitive inhibition so rare? A possible explanation, with implications for the design of drugs and pesticides. FEBS Letters 203 3–6. (10.1016/0014-5793(86)81424-7) 3720956

[bib36] Coutandin D Osterburg C Kumar Srivastav R Sumyk M Kehrloesser S Gebel J Tuppi M Hannewald J Schafer B Salah E, ***et al*** 2016 Quality control in oocytes by p63 is based on a spring-loaded activation mechanism on the molecular and cellular level. eLife 5 e13909(10.7554/elife.13909) 27021569 PMC4876613

[bib37] Cutler GB Glenn M Bush M Hodgen GD Graham CE Loriaux DL 1978 Adrenarche: a survey of rodents, domestic animals, and primates. Endocrinology 103 2112–2118.(10.1210/endo-103-6-2112) 155005

[bib38] Davis C Naci H Gurpinar E Poplavska E Pinto A Aggarwal A 2017 Availability of evidence of benefits on overall survival and quality of life of cancer drugs approved by European Medicines Agency: retrospective cohort study of drug approvals 2009–2013. BMJ 359 j4530 (10.1136/bmj.j4530) 28978555 PMC5627352

[bib40] Dalla Valle L Toffolo V Nardi A Fiore C Bernante P Di Liddo R Parnigotto PP Colombo L 2006 Tissue-specific transcriptional initiation and activity of steroid sulfatase complementing dehydroepiandrosterone sulfate uptake and intracrine steroid activations in human adipose tissue. Journal of Endocrinology 190 129–139. (10.1677/joe.1.06811) 16837617

[bib42] de Peretti E Forest MG 1976 Unconjugated dehydroepiandrosterone plasma levels in normal subjects from birth to adolescence in human: the use of a sensitive radioimmunoassay. Journal of Clinical Endocrinology and Metabolism 43 982–991. (10.1210/jcem-43-5-982) 186482

[bib39] Dehennin L Jondet M Scholler R 1987 Androgen and 19-norsteroid profiles in human preovulatory follicles from stimulated cycles: an isotope dilution-mass spectrometric study. Journal of Steroid Biochemistry 26 399–405. (10.1016/0022-4731(87)90107-5) 3586654

[bib41] Delyon J Maio M Lebbé C 2015 The ipilimumab lesson in melanoma: achieving long-term survival. Seminars in Oncology 42 387–401. (10.1053/j.seminoncol.2015.02.005) 25965357

[bib43] Derry WB Putzke AP Rothman JH 2001 *Caenorhabditis elegans* p53: role in apoptosis, meiosis, and stress resistance. Science 294 591–595. (10.1126/science.1065486) 11557844

[bib44] Dias NJ Selcer KW 2016 Steroid sulfatase in the human MG-63 preosteblastic cell line: antagonistic regulation by glucocorticoids and NFKB. Molecular and Cellular Endocrinology 420 85–96. (10.1016/j.mce.2015.11.029) 26631368

[bib45] Donehower LA Harvey M Slagle BL McArthur MJ Montgomery CA Butel JS Bradley A 1992 Mice deficient for p53 are developmentally normal but susceptible to spontaneous tumours. Nature 356 215–221. (10.1038/356215a0) 1552940

[bib46] Dranitsaris G Zhu X Adunlin G Vincent MD 2018 Cost effectiveness vs. affordability in the age of immune-oncology cancer drugs. Expert Review Pharmacoeconomic Outcomes Research 18 351–357. (10.1080/14737167.2018.1467270) 29681201

[bib47] Effron M Griner L Benirschke K 1977 Nature and rate of neoplasia found in captive wild mammals, birds, and reptiles at necropsy. Journal of the National Cancer Institute 59 185–198. (10.1093/jnci/59.1.185) 577508

[bib48] Fang X Seim I Huang Z Gerashchenko MV Xiong Z Turanov AA Zhu Y Lobanov AV Fan D Yim SH, ***et al*** 2014 Adaptations to a subterranean environment and longevity revealed by the analysis of mole rat genomes. Cell Reproduction 8 1354–1364. (10.1016/j.celrep.2014.07.030) PMC4350764 25176646

[bib50] FDA 1997 Guidance for Industry: S1B Testing for Carcinogenicity of Pharmaceuticals. Rockville, MD, USA: U.S. Department of Health and Human Services Food and Drug Administration, Center for Drug Evaluation and Research (CDER) & Center for Biologics Evaluation and Research (CBER). (available at: https://www.fda.gov/downloads/drugs/guidancecomplianceregulatoryinformation/guidances/ucm074916.pdf)

[bib49] Fenske M 1986 Adrenal function in the Mongolian gerbil (*Meriones unguiculatus*): influence of confinement stress upon glucocorticosteroid, progesterone, dehydroepiandrosterone, testosterone and androstenedione plasma levels, adrenal content and *in vitro* secretion. Experimental and Clinical Endocrinology 87 15–25. (10.1055/s-0029-1210517) 3017731

[bib51] Frank LA Rohrbach BW Bailey EM West JR Oliver JW 2003 Steroid hormone concentration profiles in healthy intact and neutered dogs before and after cosyntropin administration. Domestic Animal Endocrinology 24 43–57. (10.1016/S0739-7240(02)00204-7) 12450624

[bib52] Gassmann K Abel J Hanno Bothe H Haarmann-Stemmann T Merk HF Quasthoff KN Dino Rockel T Schreiber T Fritsche E 2010 Species-specific differential AhR expression protects human neural progenitor cells against developmental neurotoxicity of PAHs. Environmental Health Perspectives 118 1571–1577. (10.1289/ehp.0901545) 20570779 PMC2974695

[bib53] Gavrilov LA Krut’ko VN Gavrilova NS 2017 The future of human longevity. Gerontology 63 524–526.(10.1159/000477965) 28848187 PMC5654601

[bib54] Gebel J Tuppi M Krauskopf K Coutandin D Pitzius S Kehrloesser S Osterburg C Dotsch V 2017 Control mechanisms in germ cells mediated by p53 family proteins. Journal of Cell Science 130 2663–2671. (10.1242/jcs.204859) 28794013

[bib55] Gorbunova V Seluanov A Zhang Z Gladyshev VN Vijg J 2014 Comparative genetics of longevity and cancer: insights from long-lived rodents. Nature Reviews Genetics 15 531–540. (10.1038/nrg3728) PMC4353926 24981598

[bib57] Gordon GB Newitt JA Shantz LM Weng DE Talalay P 1986 Inhibition of the conversion of 3T3 fibroblast clones to adipocytes by dehydroepiandrosterone and related anticarcinogenic steroids. Cancer Research 46 3389–3395. 2939944

[bib56] Gordon MW Yan F Zhong X Mazumder PB Xu-Monette ZY Young KH Ramos KS Li Y 2005 Regulation of p53-targeting microRNAs by polycyclic aromatic hydrocarbons: implications in the etiology of multiple myeloma. Molecular Carcinogenesis 54 1060–1069. (10.1002/mc.22175) PMC4223015 24798859

[bib58] Green J Cairns J Casabonne D Wright FL Reeves G Beral V 2011 Height and cancer incidence in the Million Women Study: prospective cohort, and meta-analysis of prospective studies of height and total cancer risk. Lancet Oncology 12 785–794. (10.1016/S1470-2045(11)70154-1) 21782509 PMC3148429

[bib59] Grempler R Kienitz A Werner T Meyer M Barthel A Ailett F Sutherland C Walther R Schmoll D 2004 Tumour necrosis factor alpha decreases glucose-6-phosphatase gene expression by activation of nuclear factor kappaB. Biochemistry Journal 382 471–479. (10.1042/BJ20040160) PMC1133803 15167811

[bib60] Grube M Köck K Karner S Reuther S Ritter CA Jedlitschky G Kroemer HK 2006 Modification of OATP2B1-mediated transport by steroid hormones. Molecular Pharmacology 70 1735–1741.(10.1124/mol.106.026450) 16908597

[bib61] Gu D McNaughton L Lemaster D Lake BG Gooderham NJ Kadlubar FF Turesky RJ 2011 A comprehensive approach to the profiling of the cooked meat carcinogens 2-amino-3,8-dimethylimidazo[4,5-f]quinoxaline, 2-amino-1-methyl-6-phenylimidazo[4,5-b]pyridine, and their metabolites in human urine. Chemical Research in Toxicology 23 788–801. (10.1021/tx900436m) PMC3196554 20192249

[bib62] Guha T Malkin D 2017 Inherited *TP53* mutations and the Li-Fraumeni syndrome. Cold Spring Harbor Perspectives in Medicine 7 a026187. (10.1101/cshperspect.a026187) PMC5378014 28270529

[bib63] Guo T Chen T Gu C Li B Xu C 2015 Genetic and molecular analyses reveal G6PC as a key element connecting glucose metabolism and cell cycle control in ovarian cancer. Tumour Biology 36 7649–7658. (10.1007/s13277-015-3463-6) 25926381

[bib64] Gurven M Kaplam H 2007 Longevity among hunter-gatherers: a cross-cultural examination. Population and Development Review 33 321–365. (10.1111/j.1728-4457.2007.00171.x)

[bib65] Haccard O Dupré A Liere P Pianos A Eychenne B Jessus C Ozon R 2012 Naturally occurring steroids in Xenopus oocyte during meiotic maturation. Unexpected presence and role of steroid sulfates. Molecular and Cellular Endocrinology 362 110–119. (10.1016/j.mce.2012.05.019) 22687883

[bib66] Hattori K Yamaguchi N Umzawa K Tamura H 2012 Interferon gamma induces steroid sulfatase expression in human keratinocytes. Biological and Pharmacological Bulletin 35 1588–1593. (10.1248/bpb.b12-00028) 22975513

[bib67] Heyne K Kölsch K Bruand M Kremmer E Grässer FA Mayer J Roemer K 2015 Np9, a cellular protein of retroviral ancestry restricted to human, chimpanzee and gorilla, binds and regulates ubiquitin ligase MDM2. Cell Cycle 14 2619–2633. (10.1080/15384101.2015.1064565) 26103464 PMC4614042

[bib68] Hill K Boesch C Goodall J Pusey A Williams J Wrangham R 2001 Mortality rates among wild chimpanzees. Journal of Human Evolution 40 437–450. (10.1006/jhev.2001.0469) 11322804

[bib69] Hollstein M Sidransky D Vogelstein B Harris CC 1991 p53 mutations in human cancers. Science 253 49–53. (10.1126/science.1905840) 1905840

[bib70] Hong X Song R Song H Zheng T Wang J Liang Y Qi S Lu Z Song X Jiang H, ***et al*** 2014 PTEN antagonizes Tcl1/hnRNPK-mediated G6PD pre-mRNA splicing which contributes to hepatocarcinogenesis. Gut 63 1635–1647. (10.1136/gutjnl-2013-305302) 24352616

[bib71] Hopkins BD Goncalves MD Cantley LC 2016 Obesity and cancer mechanisms: cancer metabolism. Journal of Clinical Oncology 34 4277–4283.(10.1200/JCO.2016.67.9712) 27903152 PMC5562429

[bib72] Hubbard TD Murray IA Bisson WH Sullivan AP Sebastian A Perry GH Jablonski NG Perdew GH 2016 Divergent Ah receptor ligand selectivity during hominin evolution. Molecular Biology and Evolution 33 2648–2658. (10.1093/molbev/msw143) 27486223 PMC5026259

[bib73] Hq J Lu YX Wu QN Liu J Zeng ZL Mo H-Y Chen Y Tian T Wang Y Kang T-B, 2017 Disrupting G6PD-mediated Redox homeostasis enhances chemosensitivity in colorectal cancer. Oncogene 36 6282–6292.(10.1038/onc.2017.227) 28692052 PMC5684443

[bib74] Iafolla MAJ Juergens RA 2017 Update on Programmed Death-1 and Programmed Death Ligand-1 inhibition in the treatment of advanced metastatic non-small cell lung cancer. Frontiers in Oncology 7 67 (10.3389/fonc.2017.00067) 28428947 PMC5382272

[bib76] IARC 2015 IARC Monographs Evaluate Consumption of Red Meat and Processed Meat, Press Release Number 240. Lyon, France: International Agency for Research on Cancer (available at: https://www.iarc.fr/en/media-centre/pr/2015/pdfs/pr240_E.pdf)

[bib75] Irwin KE Greer JA Khatib J Temel JS Pirl WF 2013 Early palliative care and metastatic non-small cell lung cancer: potential mechanisms of prolonged survival. Chronic Respiratory Diseases 10 35–47. (10.1177/1479972312471549) 23355404

[bib77] Jackson K Nahata MC 2017 Rising cost of anticancer medications in the United States. Annals of Pharmacotherapy 51 706–710. (10.1177/1060028017702406) 28707550

[bib78] Janecka A Kolodziej-Rzepa M Biesaga B 2016 Clinical and molecular features of Laron Syndrome, a genetic disorder protecting from cancer. In Vivo 30 375–381. 27381597

[bib79] Jiang P Du W Mancuso A Gao X Wu M Yang X 2011 p53 regulates biosynthesis through direct inactivation of glucose-6-phosphate dehydrogenase. Nature Cell Biology 13 310–316. (10.1038/ncb2172) 21336310 PMC3110666

[bib80] Jimena P Castilla JA Peran F Ramirez JP Vergara F Jr Molina R Vergara F Herruzo A 1992 Adrenal hormones in human follicular fluid. Acta Endocrinologica 127 403–406. (10.1530/acta.0.1270403) 1471451

[bib81] Johnson IT 2017 The cancer risk related to meat and meat products. British Medical Bulletin 121 73–81. (10.1093/bmb/ldw051) 27989995

[bib82] Kabat GC Anderson ML Heo M Hosgood HD Kamensky V Bea JW Hou L Lane DS Wactawski-Wende J Manson JE, 2013 Adult stature and risk of cancer at different anatomic sites in a cohort of postmenopausal women. Cancer Epidemiology, Biomarkers and Prevention 22 1353–1363. 10.1158/1055-9965.EPI-13-0305 23887996

[bib83] Kastenhuber ER Lowe SW 2017 Putting p53 in context. Cell 170 1062–1078. (10.1016/j.cell.2017.08.028) 28886379 PMC5743327

[bib84] Kawauchi K Araki K Tobiume K Tanaka N 2008 p53 regulates glucose metabolism through an IKK-NF-kappaB pathway and inhibits cell transformation. Nature Cell Biology 10 611–618. (10.1038/ncb1724) 18391940

[bib85] Kemnitz JW Roecker EB Haffa AL Pinheiro J Kurzman I Ramsey JJ MacEwen EG 2000 Serum dehydroepiandrosterone sulfate concentrations across the life span of laboratory-housed rhesus monkeys. Journal of Medical Primatology 29 330–337. (10.1034/j.1600-0684.2000.290504.x) 11168823

[bib86] Kennedy GE 2003 Palaeolithic grandmothers? Life history theory and early ‘homo’. Journal of the Royal Anthropological Institute 9 549–572.(10.1111/1467-9655.00163)

[bib87] Kenzelmann Brož D Attardi LD 2010 *In vivo* analysis of p53 tumor suppressor function using genetically engineered mouse models. Carcinogenesis 31 1311–1318.(10.1093/carcin/bgp331) 20097732 PMC2915627

[bib89] Kim HR Roe JS Lee JE Cho EJ Youn HD 2013 P53 regulates glucose metabolism by miR-34a. Biochemical Biophysical Research Communications 437 225–231. (10.1016/j.bbrc.2013.06.043) 23796712

[bib88] Kim J Farré M Auvil L Capitanu B Larkin DM Ma J Lewin HA 2017 Reconstruction and evolutionary history of eutherian chromosomes. PNAS 114 E5379–E5388. (10.1073/pnas.1702012114) 28630326 PMC5502614

[bib90] Kopeina GS Senichkin VV Zhivotovsky B 2017 Caloric restriction – a promising anti-cancer approach: from molecular mechanisms to clinical trials. Biochimica et Biophysica Acta 1867 29–41. 27871964 10.1016/j.bbcan.2016.11.002

[bib91] Kress M May E Cassingena R May P 1979 Simian virus-40-transformed cells express new species of proteins precipitable by anti-simian virus 40 tumor serum. Journal of Virology 31 472. 225566 10.1128/jvi.31.2.472-483.1979 PMC353470

[bib93] Labrie F 2015 All sex steroids are made intracellularly in peripheral tissues by the mechanisms of intracrinology after menopause. Journal of Steroid Biochemistry and Molecular Biology 145 133–138. (10.1016/j.jsbmb.2014.06.001) 24923731

[bib94] Labrie F Bélanger A Cusan L Gomez JL Candas B 1997 Marked decline in serum concentrations of adrenal C19 sex steroid precursors and conjugated androgen metabolites during aging. Journal of Clinical Endocrinology and Metabolism 82 2396–2402. (10.1210/jcem.82.8.4160) 9253307

[bib92] Labrie F Archer DF Koltun W Vachon A Young D Frenette L Portman D Montesino M Cote I Parent J, ***et al*** 2016 Efficacy of intravaginal dehydroepiandrosterone (DHEA) on moderate to severe dyspareunia and vaginal dryness, symptoms of vulvovaginal atrophy, and of the genitourinary syndrome of menopause. Menopause 23 243–256. (10.1097/GME.0000000000000571) 30358731

[bib95] Lagunas-Rangel FA Chávez-Valencia V 2017 The curious case of the naked mole rat. Mechanisms of Ageing and Development 164 76–81. (10.1016/j.mad.2017.04.010) 28472634

[bib96] Lane DP Crawford LV 1979 T antigen is bound to a host protein in SV40 transformed cells. Nature 278 261 (10.1038/278261a0) 218111

[bib97] Laron Z Kauli R 2016 Fifty-seven years of follow-up of the Israeli cohort of Laron Syndrome patients – from discovery to treatment. Growth Hormone and IGF Research 28 53–56. (10.1016/j.ghir.2015.08.004) 26307357

[bib98] Le Pen J Laurent M Sarosiek K Vuillier C Gautier F Montessuit S Martinou JC Letai A Brawn F Juin PP 2016 Constitutive p53 heightens mitochondrial apoptotic priming and favors cell death induction by BH3 mimetic inhibitors of BCL-XL. Cell Death and Disease 7 e2083 (10.1038/cddis.2015.400) 26844698 PMC4849148

[bib99] Leroi AM Koufopanou V Burt A 2013 Cancer selection. Nature Reviews Cancer 3 226–231. (10.1038/nrc1016) 12612657

[bib100] Lettre G Kritikou EA Jaeggi M Calixto A Fraser AG Kamath RS Ahringer J Hengartner MO 2004 Genome-wide RNAi identifies p53-dependent and -independent regulators of germ cell apoptosis in *C. elegans*. Cell Death and Differentiation 11 1198–1203. (10.1038/sj.cdd.4401488) 15272318

[bib101] Linzer DI Levine AJ 1979 Characterization of a 54K Dalton cellular SV40 tumor antigen present in SV40-trnsformed cells and uninfected embryonal carcinoma cells. Cell 17 43 (10.1016/0092-8674(79)90293-9) 222475

[bib102] Lombard LS Witte EJ 1959 Frequency and types of tumors in mammals and birds of the Philadelphia Zoological Garden. Cancer Research 19 127–141. 13629476

[bib103] Loukopoulos P Thornton JR Robinson WF 2003 Clinical and pathological relevance of p53 index in canine osseous tumors. Veterinary Pathology 40 237–248. (10.1354/vp.40-3-237) 12724563

[bib104] Lozano G Liu G 1998 Mouse models dissect the role of p53 in cancer and development. Seminars in Cancer Biology 8 337–344. (10.1006/scbi.1998.0096) 10101799

[bib105] Ma J Ward EM Siegel RL Jemal A 2015 Temporal trends in mortality in the United States, 1969–2013. JAMA 314 1731–1739. 26505597 10.1001/jama.2015.12319

[bib106] Malvezzi M Carioli G Bertuccio P Bofetta P Levi F 2017 European cancer mortality predictions for the year 2017, with focus on lung cancer. Annals of Oncology 28 1117–1123. (10.1093/annonc/mdx033) 28327906

[bib107] Mangiapane E Pessione A Pessione E 2014 Selenium and selenoproteins: an overview on different biological systems. Current Protein and Peptide Science 14 1–10. 10.2174/1389203715666140608151134 24910086

[bib108] McClure HM 1973 Tumors in nonhuman primates: observations during a six-year period in the Yerkes primate center colony. American Journal of Physical Anthropology 38 425–429. (10.1002/ajpa.1330380243) 4347670

[bib109] McCorkle R Strumpf NE Nuamah IF Adler DC Cooley ME Jepson C Lusk EJ Torosian M 2000 A specialized home care intervention improves survival among older post-surgical cancer patients. Journal of the American Geriatric Society 48 1707–1713. (10.1111/j.1532-5415.2000.tb03886.x) 11129765

[bib110] McDermott D Lebbé C Hodi FS Maio M Weber JS Wolchok JD Thompson JA Balch CM 2014 Durable benefit and the potential for long-term survival with immunotherapy in advanced melanoma. Cancer Treat Reviews 40 1056–1064. (10.1016/j.ctrv.2014.06.012) 25060490

[bib111] McHenry HM 1976 Early hominid body weight and encephalization. American Journal of Physical Anthropology 45 77–83. (10.1002/ajpa.1330450110)

[bib112] Merkel O Taylor N Prutsch N Staber PB Moriggl R Turner SD Kenner L 2017 When the guardian sleeps: reactivation of the p53 pathway in cancer. Mutation Research 773 1–13. (10.1016/j.mrrev.2017.02.003) 28927521

[bib113] Mialot JP Thibier M Toublanc JE Castanier M Scholler R 1988 Plasma concentration of luteinizing hormone, testosterone, dehydroepiandrosterone, androstenedione between birth and one year in the male dog: longitudinal study and hCG stimulation. Andrologia 20 145–154. (10.1111/j.1439-0272.1988.tb00678.x) 2968771

[bib114] Milton K 1999 A hypothesis to explain the role of meat-eating in human evolution. Evolutionary Anthropology 8 11–21. (10.1002/(SICI)1520-6505(1999)8:1<11::AID-EVAN6>3.0.CO;2-M)

[bib115] Mongillo P Prana E Gabai G Bertotto D Marinelli L 2014 Effect of age and sex on plasma cortisol and dehydroepiandrosterone concentrations in the dog (*Canis familiaris*). Research in Veterinary Science 96 33–38. (10.1016/j.rvsc.2013.10.010) 24269080

[bib116] Nakamura Y Gang HX Suzuki T Sasano H Rainey WE 2009 Adrenal changes associated with adrenarche. Reviews in Endocrine and Metabolic Disorders 10 19–26. (10.1007/s11154-008-9092-2) 18821019 PMC3712864

[bib117] Napoli M Flores ER 2016 The p53 family orchestrates the regulation of metabolism: physiological regulation and implications for cancer therapy. British Journal of Cancer 116 149–155. (10.1038/bjc.2016.384) 27884017 PMC5243983

[bib120] Nyce JW 2017 Species-specificity of an adrenal androgen-mediated kill switch that is triggered by the inactivation of the p53 tumor suppressor. Translational Medicine Reports 1 76–85. (10.4081/tmr.6773)

[bib121] Odell WD Parker LN 1985 Control of adrenal androgen production. Endocrine Research 10 617–630. (10.1080/07435808409036520) 6100259

[bib122] Ogden CL Carroll MD 2010 Prevalence of Overweight, Obesity, and Extreme Obesity among Adults: United States, Trends 1960–1962 through 2007–2008. Hyattsville, MD, USA: National Center for Health Statistics (available at: https://www.cdc.gov/nchs/data/hestat/obesity_adult_07_08/obesity_adult_07_08.pdf)

[bib123] Olivier M Hollstein M Hainaut P 2010 TP53 mutations in human cancers: origins, consequences, and clinical use. Cold Spring Harbor Perspectives in Biology 2 a001008. 20182602 10.1101/cshperspect.a001008 PMC2827900

[bib124] Olshansky AJ Carnes BA Cassel C 1990 In search of Methuselah: estimating the upper limits to human longevity. Science 250 634–640. (10.1126/science.2237414) 2237414

[bib125] Ong J-S An J Law M Whiteman DC Neale RE Gharahkhani P MacGregor S 2018 Height and overall cancer risk and mortality: evidence from a Mendelian randomization study on 310,000 UK Biobank participants. British Journal of Cancer 118 1262–1267. (10.1038/s41416-018-0063-4) 29581483 PMC5943400

[bib126] Orentreich N Brind JL Rizer RL Vogelman JH 1984 Age changes and sex differences in serum dehydroepiandrosterone sulfate concentrations throughout adulthood. Journal of Clinical Endocrinology and Metabolism 59 551–555. (10.1210/jcem-59-3-551) 6235241

[bib127] Pankow S Bamberger C 2007 The p53 tumor suppressor-like protein nvp63 mediates selective germ cell death in the sea anemone *Nematostella vectensis*. PLoS ONE 2 e782 (10.1371/journal.pone.0000782) 17848985 PMC1964547

[bib128] Papadopoulos D Shihan M Scheiner-Bobis G 2017 Physiological implications of DHEAS-induced non-classical steroid hormone signaling. Journal of Steroid Biochemistry and Molecular Biology 179 73–78. (10.1016/j.jsbmb.2017.10.002) 29017935

[bib129] Parker CR 1999 Dehydroepiandrosterone and dehydroepiandrosterone sulfate production in the human adrenal during development and aging. Steroids 64 640–647.(10.1016/S0039-128X(99)00046-X) 10503722

[bib130] Parker LN Odell WD 1980 Control of adrenal androgen secretion. Endocrine Reviews 1 392–410. (10.1210/edrv-1-4-392) 6265199

[bib131] Pashen RL Sheldrick EL Allen WR Flint AP 1982 Dehydroepiandrosterone synthesis by the fetal foal and its importance as an oestrogen precursor. Journal of Reproduction and Fertility Supplement 32 389–397. 6220147

[bib132] Perez-Fernandez R Facchinetti F Beiras A Lima L Gaudiero GJ Genazzani AR Devesa J 1987 Morphological and functional stimulation of adrenal reticularis zone by dopaminergic blockade in dogs. Journal of Steroid Biochemistry 28 465–470.(10.1016/0022-4731(87)90503-6) 2824928

[bib133] Perkins SN Hursting SD Haines DC James SJ Miller BJ Phang JM 1997 Chemoprevention of spontaneous tumorigenesis in nullizygous p53-deficient mice by dehydroepiandrosterone and its analog 16-alpha-fluoro-5-androsten-17-one. Carcinogenesis 18 989–994.(10.1093/carcin/18.5.989) 9163685

[bib134] Perret M Aujord F 2005 Aging and season affect plasma dehydroepiandrosterone sulfate (DHEA-S) levels in a primate. Experimental Gerontology 40 582–587.(10.1016/j.exger.2005.05.002) 16019179

[bib135] Petitjean A Achatz MI Borresen-Dale AL Hainaut P Olivier M 2007 TP53 mutations in human cancers: functional selection and impact on cancer prognosis and outcomes. Oncogene 26 2157–2165.(10.1038/sj.onc.1210302) 17401424

[bib136] Pieper DR Lobocki CA 2000 Characterization of serum dehydroepiandrosterone secretion in golden hamsters. Proceedings of the Society for Experimental Biology and Medicine 224 278–284. (10.1046/j.1525-1373.2000.22432.x) 10964263

[bib137] Prasad V Mailankody S 2017 Research and development spending to bring a single cancer drug to market and revenues after approval. JAMA Internal Medicine 177 1569–1575. 28892524 10.1001/jamainternmed.2017.3601 PMC5710275

[bib138] Quinn TA Ratnayake U Dickinson H Nguyen TH McIntosh M Castillo-Melendez M Conley AJ Walker DW 2013 Ontogeny of the adrenal gland in the spiny mouse, with particular reference to production of the steroids cortisol and dehydroepiandrosterone. Endocrinology 154 1190–1201. (10.1210/en.2012-1953) 23354096

[bib139] Raeside JI Renaud RL Christie HL 1997 Postnatal decline in gonadal secretion of dehydroepiandrosterone and 3â-hydroxyandrosta-5,7-dien-17-one in the newborn foal. Journal of Endocrinology 155 277–282. (10.1677/joe.0.1550277) 9415062

[bib140] Rainey WE Rehman KS Carr BR 2004 The human fetal adrenal: making adrenal androgens for placental estrogens. Seminars in Reproductive Medicine 22 327–336. (10.1055/s-2004-861549) 15635500

[bib141] Ratcliffe HL 1933 Incidence and nature of tumors in captive wild mammals and birds. American Journal of Cancer 17 116–135. (10.1158/ajc.1933.116)

[bib142] Rege J Rainey WE 2012 The steroid metabolome of adrenarche. Journal of Endocrinology 214 133–143. (10.1530/JOE-12-0183) 22715193 PMC4041616

[bib143] Rondelli MC Munhoz TD Catandi PB Freschi CR Palacios Junior RJ Machado RZ Tinucci-Costa M 2015 Serum DHEA-S increases in dogs naturally infected with *Ehrlichia canis*. Research in Veterinary Science 100 18–20. (10.1016/j.rvsc.2015.04.010) 25956636

[bib144] Roser M 2018 Life expectancy. Oxford, UK: Our World in Data (available at: https://ourworldindata.org/life-expectancy)

[bib145] Ryan KM 2011 p53 and autophagy in cancer: guardian of the genome meets guardian of the proteome. European Journal of Cancer 47 44–50. (10.1016/j.ejca.2010.10.020) 21112207

[bib146] Schiebinger RJ Albertson BD Barnes KM Cutler GB Jr Loriaux DL 1981 Developmental changes in rabbit and dog adrenal function: a possible homologue of adrenarche in the dog. American Journal of Physiology 240 E694–E699. 6264794 10.1152/ajpendo.1981.240.6.E694

[bib147] Schilling MM Oeser JK Chandy JK Flemming BP Allen SR O’Brien RM 2008 Sequence variation between the mouse and human Glucose-6-Phosphatase Catalytic Subunit gene promoters results in differential activation by Peroxisome Proliferator Activator Receptor Gamma Coactivator-1alpha. Diabetologia 51 1505–1514. (10.1007/s00125-008-1050-8) 18563384 PMC2590337

[bib148] Schuler G Dezhkam Y Bingsohn L Hoffmann B Failing K Galuska CE Hartmann MF Sanchez-Guijo A Wudy SA 2014 Free and sulfated steroids secretion in postpubertal boars (*Sus scrofa domestica*). Reproduction 148 303–314. (10.1530/REP-14-0193) 24961601

[bib149] Schulz S Nyce JW 1991 Inhibition of protein isoprenylation and p21ras membrane association by dehydroepiandrosterone in human colonic adenocarcinoma cells. Cancer Research 51 6563–6567. 1835900

[bib150] Schumacher B Hoffmann K Boulton S Gartner A 2001 The *C. elegans* homolog of the p53 tumor suppressor is required for DNA damage-induced apoptosis. Current Biology 11 1722–1727. (10.1016/S0960-9822(01)00534-6) 11696333

[bib151] Schwartzenberg-Bar-Yoseph F Armoni M Karnieli E 2004 The tumor suppressor p53 down-regulates glucose transporters GLUT1 and GLUT4 gene expression. Cancer Research 64 2627–2633. (10.1158/0008-5472.CAN-03-0846) 15059920

[bib152] Sen T Sen N Huang Y Sinha D Luo ZG Ratovitski E Sidransky D 2011 Tumor protein p63/nuclear factor kappa-B feedback loop in regulation of cell death. Journal of Biological Chemistry 286 43204–43213. (10.1074/jbc.M111.257105) 22020940 PMC3234803

[bib153] Shen L Sun X Fu Z Yang G Li J Yao L 2012 The fundamental role of the p53 pathway in tumor metabolism and its implication in tumor therapy. Clinical Cancer Research 18 1561–1567. (10.1158/1078-0432.CCR-11-3040) 22307140

[bib154] Shams I Malik A Manov I Joel A Band M Avivi A 2013 Transcription pattern of p53-targeted DNA repair genes in the hypoxia-tolerant subterranean mole rat Spalax. Journal of Molecular Biology 425 1111–1118. (10.1016/j.jmb.2013.01.007) 23318952

[bib155] Siddiqui M Rajkumar SV 2012 The high cost of cancer drugs and what we can do about it. Mayo Clinic Proceedings 87 935–943. (10.1016/j.mayocp.2012.07.007) 23036669 PMC3538397

[bib156] Smith DW 1993 Human Longevity. Oxford, UK: Oxford University Press.

[bib157] Sorwell KG Urbanski HF 2013 Causes and consequences of age-related steroid hormone changes: insights gained from nonhuman primates. Journal of Neuroendocrinology 25 1062–1069. (10.1111/jne.12064) 23796387 PMC3883982

[bib158] Speidel D 2010 Transcription-independent p53 apoptosis: an alternative route to death. Trends in Cell Biology 20 14–24. (10.1016/j.tcb.2009.10.002) 19879762

[bib159] Strano S Rossi M Fontemaggi G Munarriz E Soddu S Sacchi A Blandino G 2001 From p63 to p53 across p73. FEBS Letters 490 163–170. (10.1016/S0014-5793(01)02119-6) 11223031

[bib160] Suh EK Yang A Kettenbach A Bamberger C Michaelis AH Zhu Z Elvin JA Bronsom RT Crum CP McKeon F 2006 p63 protects the female germ line during meiotic arrest. Nature 444 624–628. (10.1038/nature05337) 17122775

[bib161] Sulak M Fong L Mika K Chigurupati S Yon L Mongan NP Emes RD Lynch VJ 2016 TP53 copy number expansion is associated with the evolution of increased body size and an enhanced DNA damage response in elephants. eLife 5 e11994. 27642012 10.7554/eLife.11994 PMC5061548

[bib162] Sulcová J Hill M Hampl R Stárka L 1997 Age and sex related differences in serum levels of unconjugated dehydroepiandrosterone and its sulphate in normal subjects. Journal of Endocrinology 154 57–62. (10.1677/joe.0.1540057) 9246938

[bib163] Takahashi T Nau MM Chiba I Birrer MJ Rosenberg RK Vinocour M Levitt M Pass H Gazdar AF Minna JD 1989 p53: a frequent target for genetic abnormalities in lung cancer. Science 246 491–494. (10.1126/science.2554494) 2554494

[bib164] Tang J Ning R Zeng B Li Y 2016 Molecular evolution of PTEN pseudogenes in mammals. PLoS ONE 11 e0167851 (10.1371/journal.pone.0167851) 27936183 PMC5148010

[bib165] Tian X Azpurua J Hine C Vaidya A Myakishev-Rempel M Ablaeva J Mao Z Nevo E Gorbunova V Seluanov A 2013 High-molecular-mass hyaluronan mediates the cancer resistance of the naked mole rat. Nature 499 346–349. (10.1038/nature12234) 23783513 PMC3720720

[bib166] Tollis M Schiffman JD Boddy AM 2017 Evolution of cancer suppression as revealed by mammalian comparative genomics. Current Opinions in Genetics and Development 42 40–47. (10.1016/j.gde.2016.12.004) 28161621

[bib167] Tremblay Y Belanger A 1985 Changes in plasma steroid levels after single administration of hCG or LHRH agonist analogue in dog and rat. Journal of Steroid Biochemistry 22 315–320. (10.1016/0022-4731(85)90432-7) 2581068

[bib168] Tretyakova N Matter B Jones R Shallop A 2002 Formation of benzo[a]pyrene diol epoxide-DNA adducts at specific guanines within K-ras and p53 gene sequences: stable isotope-labeling mass spectrometry approach. Biochemistry 41 9535–9544. (10.1021/bbib25540i) 12135376

[bib169] Trinkaus E 2011 Late pleistocene adult mortality patterns and modern human establishment. PNAS 108 1267–1271. (10.1073/pnas.1018700108) 21220336 PMC3029716

[bib170] van Weerden WM Bierings HG van Steenbrugge GJ de Jong FH Schröder FH 1992 Adrenal glands of mouse and rat do not synthesize androgens. Life Science 50 857–861. (10.1016/0024-3205(92)90204-3) 1312193

[bib171] Varley JM 2003 Germline TP53 mutations and Li-Fraumeni syndrome. Human Mutation 21 313–320. (10.1002/humu.10185) 12619118

[bib172] Vermuelen A 1980 Adrenal androgens and aging. In Adrenal Androgens, pp 207–217. Eds Genazzani AR, Thijssen JHH & Siiteri PK New York, NY, USA: Raven Press.

[bib174] Wang TT Hursting SD Perkins SN Phang JM 1997 Effects of dehydroepiandrosterone and calorie restriction on the Bcl-2/Bax-mediated apoptotic pathway in p53-deficient mice. Cancer Letters 116 61–69. (10.1016/S0304-3835(97)00175-4) 9177459

[bib173] Wang T Zeng J Lowe CB Sellers RG Salama SR Yang M Burges SM Brachmann RK Haussler D 2007 Species-specific endogenous retroviruses shape the transcriptional network of the human tumor suppressor protein p53. PNAS 104 18613–18618. (10.1073/pnas.0703637104) 18003932 PMC2141825

[bib175] Warner GJ Berry MJ Moustafa ME Carlson BA Hatfield DL Faust JR 2000 Inhibition of selenoprotein synthesis by selenocysteine tRNA[Ser]Sec lacking isopentenyladenosine. Journal of Biological Chemistry 275 28110–28119. 10821829 10.1074/jbc.M001280200

[bib178] Weiss KM 1981 Evolutionary perspectives on human aging. In Other Ways of Growing Old, pp 25–58. Eds Amoss & Harrell S Stanford, CA, USA: Stanford University Press.

[bib177] Weiss KM 1984 On the number of members of the Genus Homo who have ever lived, and some evolutionary implications. Human Biology 56 637–649. 6442261

[bib176] Weisz L Damalas A Liontos M Karakaidos P Fontemaggi G Maor-Aloni R Kalis M Levrero M Strano S Gorgoulis VG, 2007 Mutant p53 enhances nuclear factor KB activation by tumor necrosis factor alpha in cancer cells. Cancer Research 67 2396–2401. (10.1158/0008-5472.CAN-06-2425) 17363555

[bib180] Yang HC Wu YH Liu HY Stern A Chiu DT 2016 What has passed is prolog: new cellular and physiological roles of G6PD. Free Radical Research 50 1047–1064. (10.1080/10715762.2016.1223296) 27684214

[bib181] Yin Y Shen WH 2008 PTEN – a new guardian of the genome. Oncogene 27 5443–5453. (10.1038/onc.2008.241) 18794879

[bib182] Yokokawa T Sato K Iwanaka N Honda M Higashida K Lemitsu M Hashimoto T 2015 Dehydroepiandrosterone activates AMP kinase and regulates GLUT4 and PGC-1alpha expression. Biochemical Biophysical Research Communications 463 42–47.(10.1016/j.bbrc.2015.05.013) 25983323

[bib183] Yoneyama A Kamiya Y Kawaguchi M Fujinami T 1997 Effects of dehydroepiandrosterone on proliferation of human aortic smooth muscle cells. Life Science 60 833–838. (10.1016/S0024-3205(97)00011-8) 9076322

[bib184] Yue X Zhao Y Xu Y Zheng M Feng Z Hu W 2017 Mutant p53 in cancer: accumulation, gain-of-function, and therapy. Journal of Molecular Biology 429 1595–1606. (10.1016/j.jmb.2017.03.030) 28390900 PMC5663274

[bib185] Zhang P Tu B Wang H Cao Z Tang M Zhang C Gu B Li Z Wang L Yang Y, ***et al*** 2014 Tumor suppressor p53 cooperates with SIRT6 to regulate gluconeogenesis by promoting FoxO1 nuclear exclusion. PNAS 111 10684–10689. (10.1073/pnas.1411026111) 25009184 PMC4115576

[bib186] Zhou M Luo J Chen M Yang H Learned RM DePaoli AM Tian H Ling L 2017 Mouse species-specific control of hepatocarcinogenesis and metabolism by FGF19/FGF15. Journal of Hepatology 66 1182–1192. (10.1016/j.jhep.2017.01.027) 28189755

[bib187] Zhuang W Tajima S Okada K Ikawa Y Aidaq Y 1997 Point mutation of p53 tumor suppressor gene in bovine leukemia virus-induced lymphosarcoma. Leukemia 11 (Supplement 3) 344–346. 9209385

