# Supplementary material for: Detection of a novel, primate-specific ‘kill switch’ tumor suppression mechanism that may fundamentally control cancer risk in humans: an unexpected twist in the basic biology of TP53
Source: Endocr Relat Cancer. 2018 Jun 25;25(11):R497–517. doi: 10.1530/ERC-18-0241 (PMC6106910; doi:10.1530/ERC-18-0241)
Supplement: Supplementary Section 1 [file erc-25-R497-s001.pdf]

Supplemental Section 1

Of mice and men and dogs and fire

Dogs have a rudimentary form of the primate-specific kill switch, made possible by circulating DHEAS. Even though the levels of circulating DHEAS in canines are only a tiny fraction of those observed in primates (Perez-Fernandez et al., 1987; Schiebinger *et al*., 1981; Cutler *et al*., 1978), they are more than an order of magnitude higher than those observed in rodents [Figure 3 in main article]. How is it that dogs thus appear to have copied at least some facet of the kill switch tumor suppressor system employed by humans? One answer to this question might be that circulating DHEAS served multiple important functions in the evolutionary trajectories of both primates and dogs, and that these functions addressed problems that might otherwise have prevented a relationship to develop between these species. Thus, DHEA opposes cortisol in the HPA axis, and it has been suggested that such inhibition of the fight or flight response may have enabled early humans to live together in larger and larger groups (Campbell, 2006; Suzuki *et al*., 2004). Chimpanzees and bonobos live in troops of up to 80 individuals, an effective strategy to defend against their primary predator, leopards (D'Amour *et al*., 2006). Given that much larger felines dominated the landscape during human evolution (Camaros *et al*., 2016), group habitation was clearly one of several environmental adaptations that was enabling for the survival of our species as well. As in *Homo sapiens* and our predecessor, *Homo erectus*, circulating DHEAS may have enabled much more complex social interaction among wolves than is observed in other species that live in groups. We have suggested that circulating DHEAS, and a similar inhibition of the fight or flight response, may have selected wolves for domestication that had increased levels of circulating DHEAS (Nyce, 2017). Put more precisely, circulating DHEAS and its moderating effects upon the HPA axis *in both species* may have permitted the coming together of man and dog. The sharing of a similar tumor suppression mechanism based upon circulating DHEAS may also have enabled this inter-species relationship as it faced many challenges, one of which was

species-specific carcinogen exposure subsequent to the harnessing of fire by man. Nevertheless, the levels of circulating DHEAS in the dog are a fraction of what they are in *Strepsirrhine* primates, let alone *Haplorrhine* primates such as *Homo sapiens* (Main article Figure 3). Furthermore, while canines do not have the ACAG tetranucleotide of laboratory mice and rats that prevents the accumulation of G6P and therefore kill switch function, neither do they have the *Haplorrhine* primate-specific GAAT tetranucleotide that enables G6P accumulation in the presence of DHEA. Rather, canines have the canonical GCAG tetranucleotide characteristic of most species, and it is not yet known what effect this tetra-nucleotide has on PGC-1alpha-mediated regulation of G6PC activity (Figure 2, main article).

The pyrophilic primate

About 400,000 years ago, our immediate ancestor, *Homo erectus*, harnessed fire as a tool for survival. This period in Africa is known to have been characterized by extreme and rapid fluctuations between closed canopy forests, woodland, and grasslands, which changed the fire regime of the region and increased the occurrence of natural fires. The “pyrophilic primate” hypothesis proposes that human fire dependence was the result of adapting to progressively fire-prone environments (Parker *et al.*, 2016). Before humans learned to make fire at will, they may have captured natural fire and brought it into their habitats where it could be shielded from the elements and thereby continuously maintained. Fire, of course, was highly adaptive for humans as it improved the energy value of food by cooking, provided physical warmth, and offered a shield against fire-wary feline predators. The habitats of fire-dependent *Homo erectus* and subsequent *Homo sapiens* throughout almost all of their existence as species would thus have been characterized by extremely high levels of polycyclic aromatic hydrocarbons (PAHs) and other mutagenic toxins produced in the incomplete combustion of organic matter. Such exposure would have been far in excess of anything that we can imagine in the modern world, and would have been constant and life-long. PAHs are mutagenic (Aoki, 2017), carcinogenic

(Kim *et al*., 2013), cytotoxic (Lutz *et al*., 1998) and teratogenic (Liu *et al*., 2014). In particular, the toxicity to oocytes and spermatozoa of benzo(a)pyrene (B(a)P), a major PAH produced in the incomplete combustion of wood and other biomaterials, is profound (Zhang *et al*., 2017; O'Brien *et al*., 2016; Fowler *et al*., 2014; Einaudi *et al*., 2014 Ge *et al*., 2012; Kee *et al*, 2010; Mattison *et al*., 1983). We have suggested that the uncompetitive kinetics of DHEA inhibition with respect to G6PD evolved as part of a kill switch system that optimized reproductive fitness by eliminating germ cells with damaged genomes. As described in the main article, such a kill switch mechanism may utilize the large DHEAS pools that are specific to gonadal tissue to effect irreversible uncompetitive inhibition of G6PD by DHEA exclusively in germ cells, e.g. oocytes (Haccard *et al*., 2012), with compromised genomes. Primates subsequently evolved a separate somatic cell kill switch mechanism that was enabled by high circulating levels of DHEAS, and mutations in Glucose-6-phosphatase (G6PC) that permitted accumulation of Glucose-6-phosphate (see Figure 2 in the main article).

While we envision that the germ cell kill switch mechanism employing gonadal DHEAS played an important role in maintaining reproductive fitness in the presence of the extreme PAH levels in smoke-filled primitive human habitats, the somatic cell kill switch mechanism may have maintained somatic tissues in a state also required for successful reproduction. As noted above, B(a)P is an especially powerful mutagen and carcinogen. It is capable of inducing cellular transformation in most tissues of the body, but especially the lung after aerosolized exposure. The primate-specific kill switch may have enabled early humans to survive long enough to reproduce in habitats characterized by extreme levels of exposure to aerosolized B(a)P that became part of their daily life after becoming dependent upon fire. In rats, exposure to aerosolized B(a)P and other PAH for 17 hours per day, 5 days per week, for 20 months produced lung tumors in 97% of animals (Heinrich et al., 1994). Prehistoric humans exposed to aerosolized B(a)P and other PAH for decades longer periods of time than the rodents in this experiment may have avoided a similar fate by the circulating DHEAS and

G6PC promoter mutations that enabled the kill switch function to operate in them. Similarly, as discussed in the main article, the processing of food using heat produces a variety of mutagenic and carcinogenic substances which are distributed widely throughout the body after eating. The adrenal androgen-mediated kill switch tumor suppressor mechanism, distributed among all somatic cells, may have enabled the evolutionary trajectory of our species subsequent to the harnessing of fire.

Primate origin of the kill switch

Humans may not have been able to successfully harness fire and simultaneously maintain somatic fitness in smoke-filled habitats, eating carcinogen-laced, heat-processed meat, if ancestral primates had not already evolved circulating DHEAS and G6PC promoter mutations that permitted accumulation of G6P. The question arises, then, what environmental pressure selected for these evolutionary responses specific for the *Haplorrhine* lineage prior to the harnessing of fire by *Homo* species? Although there remains considerable debate regarding the origin of primates, most accounts place it at about 80-90 MYA (Perelman *et al*., 2011; Goodman *et al*., 1998). Euprimates, the oldest primates of modern aspect, include anthropoid *Haplorrhines* and prosimian *Strepsirrhines*. Both the anthropoid and the prosimian lineages appear abruptly together in the Paleo Eocene Thermal Maximum (PETM) at the beginning of the Eocene, about 56 million years ago (Dunn *et al*., 2016; Ni *et al*., 2013; Miller *et al*., 2005). Circulating DHEAS is found in both *Strepsirrhines* and *Haplorrhines*, indicating that this feature was a primordial element of primate evolution (Blevins *et al*., 2013; Bernstein *et al*., 2012; Perret and Aujard, 2005). But G6PC mutations conferring adrenal androgen-mediated kill switch function came later, as they occur only in the *Haplorrhines* and not the *Strepsirrhines.* Among *Haplorrhines*, both Old World *Catarrhines* (e.g. Sooty Mangabey, Chimpanzee) and New World *Platyrrhines* (e.g. marmoset) demonstrate these G6PC promoter mutations. (See Figure 2 in the main article).

Why did such an adrenal androgen-mediated kill switch evolve in *Haplorrhines*? Clearly, the *Haplorrhine*-specific nature of these G6PC promoter mutations suggests that they were enabling for the evolution of this lineage. PAH are also found in fruits and cereals (Phillips, 1999), and the evolution of a longer lifespan in vegetarian Haplorrhine primates may have already required a counter to the presence of this carcinogen. But environmental influences may have played the greatest role in the evolution of the kill switch tumor suppression system. Thus, the PETM was one of the most dramatic periods of rapid global warming in the geological record, corresponding to an abrupt negative 3-4% carbon isotope excursion in both marine and terrestrial sediments (Thomas *et al*., 2002). It is generally believed that increased levels of combustion of organic matter worldwide, i.e., wildfires, contributed to this global warming (Denis *et al*., 2017; Selkin *et al*., 2015). PAH produced by such wildfires appear to have contaminated the worldwide environment in which the *Haplorrhines* evolved (Denis *et al*., ibid.; Robson *et al*., 2014), and one can reasonably speculate that kill switch function provided a selective advantage to this lineage. PAH contamination during the PETM would have entered the lungs of terrestrial animals both by inhalation, and as a contaminant of food resources that contained PAH Li *et al*., 2017; Wang *et al*., 2017). It is also possible that omnivorous, diurnal *Haplorrhine* euprimates may have purposefully selected fire-combusted (and therefore PAH-contaminated) landscapes as productive places to forage for food, perhaps attracted by aromatic cues from large insects and small animals that had been unable to escape wild fires. We speculate that such behavior, which would require some understanding of fire, may have been outside the range of much smaller-brained, nocturnal *Strepsirrhine* euprimates, but perhaps not the larger brained *Haplorrhines*. Modern Chimpanzees show conceptualization of fire behavior (Pruetz and LaDuke, 2010). Although no such foraging behavior by *Haplorrhine* euprimates at fire-combusted landscapes has yet been reported despite detailed observation (Goodall, 1986), the fire regime of most modern primate landscapes is dramatically less incendiary compared

to that which existed during the PETM. The exception to this is the fire-ravaged environments of orangutans in Indonesia, which are currently threatening the existence of this endangered species. Whatever the details of the ecological niches existing during the PETM, the kill switch mechanism enabled by G6PC promoter mutations specific for *Haplorrhines*, and by the circulating DHEAS characteristic of all primates, may have provided the selective advantage the *Haplorrhine* lineage needed to successfully adapt to the PAH-contaminated landscape of that era, and to enable subsequent *Homo* species to successfully harness fire as a survival tool.

DHEAS may have enabled canine domestication not only by acting as a force to counter the fight or flight response, but also by acting to protect dogs from the species-specific carcinogen exposure that occurred by their co-habitation with humans. Without their rudimentary kill switch, dogs may not have been able to join us in our smoke-filled habitats, or consume the heat-processed meat that humans offered to them. The fact that canine domestication occurred relatively recently (Botigue *et al*., 2017), and that hydrocarbon exposure is no longer the selective pressure that it once was for dogs, may explain why their kill switch remains rudimentary.

PAHs are also neurotoxins

The evolution of circulating DHEAS and the mutations required in the G6PC gene to enable kill switch function are not the only species-specific alterations that may have been required to enable Man's harnessing of fire. In addition to being mutagens, PAHs are potent neurotoxins in most species, a toxicity mediated in large measure by their binding to the Aryl Hydrocarbon Receptor (AHR). Wild fires were ubiquitous in prehistoric nature, usually caused by lightning strikes, and PAHs also exist as part of the natural environment, for example from geological processes that break down organic matter to produce oil and tar, and even in fruit and cereal grains, as noted above. In fact, PAH have been identified in space, and most of the carbon in the universe is thought to be sequestered in PAHs. All creatures are thus exposed to PAH. When humans turned fire into a tool,

however, they initiated a level of PAH exposure unprecedented in the animal kingdom. AHR evolved in diverse animals as a natural sensor of exogenous toxins such as PAH; linked to appropriate neurocircuits, AHRs could be used to avoid their ingestion. Such avoidance was appropriate, because in mice and rats and other studied species, developmental neurotoxicity of PAH exposure appears to be mediated by AHR, both *in utero* and after. Since primitive humans utilizing fire were exposed to much larger doses of PAHs than other animals were, and the main thrust of human evolution was based upon enhanced brain function, developmental neurotoxicity would clearly have reduced reproductive fitness. It is thus of interest that while PAH induce developmental neurotoxicity mediated by AHR in mice and rats, humans display a species-specific loss of AHR expression in the progenitor neuronal cells of fetal and childhood development (Gassmann *et al*., 2010) Adaptations in AHR appear also to be unique in modern humans as compared to Neanderthals and non-human primates (Hubbard *et al*., 2016). *Homo sapiens* are thus characterized as extraordinarily resistant to the AHR-mediated developmental neurotoxicity that affects other species exposed to PAH. Along with circulating DHEAS and mutations in the G6PC promoter, modification of AHR may be another adaptation that was required in the successful harnessing of fire. Although humans no longer live in smoke-filled caves, we continue to harness various improvements upon fire to exploit our environment, an adaptation which continues to distinguish our species from all others. We are also the only species which purposefully infuses our lungs with PAHs for pleasure.

References for Supplemental Text 1

Aoki Y 2017 Evaluation of *in vivo* mutagenesis for assessing the health risk of air pollutants. *Genes and Environment* **39**:16-20. (https://genesenvironment.biomedcentral.com/articles/10.1186/s41021-016-0064-6)

Bernstein RM, Sterner KN, Wildman DE 2012 Adrenal Androgen Production in Catarrhine Primates and the Evolution of Adrenarche. *American Journal of Physical Anthropology* **147**(3): 389– 400.   (https://www.ncbi.nlm.nih.gov/pmc/articles/PMC4469270/)

Blevins JK, Coxworth JE, Herndon JG, Hawkes K 2013 Brief communication: Adrenal androgens and aging: Female chimpanzees (*Pan troglodytes*) compared with women. *American Journal of Physical Anthropology* **151**(4):643-648. (https://www.ncbi.nlm.nih.gov/pmc/articles/PMC4412270/)

Botigué LR, Song S, Scheu A, Gopalan S, Pendleton AL, Oetjiens M, Taravella AM, Seregely T, Zeeb-Lanz A, Arbogast RM *et al.*, 2017 Ancient European dog genomes reveal continuity since the Early Neolithic. *Nature Communications* **8**:16082. (https://www.ncbi.nlm.nih.gov/pmc/articles/PMC5520058/)

Camaros E, Cueto M, Lorenzo C, Villaverde V, Rivals F 2016 Large carnivore attacks on hominins during the Pleistocene: a forensic approach with a Neanderthal example. *Archaeological and Anthropological Sciences* **8**(3):635-646. (https://link.springer.com/epdf/10.1007/s12520-015-0248-1?shared_access_token=GOH4sXFinali8dqQKlugQve4RwlQNchNByi7wbcMAY7NwpEFFKLN38MF3PBkL7I7zqAGcyA0-cw6dhm-A2zLQDzemiEpNKBWEi1ULh3EVRUpXVsWY6EiucdT9iQGUE8tZjBxZ-J3uWwy8EQaa23PARo9ESwfVb7tyuBsTrqOUtY=)

Campbell B 2006 Adrenarche and the evolution of human life history *American Journal of Human Biology* **18**(5):569-589. (https://www.ncbi.nlm.nih.gov/pubmed/?term=Adrenarche+and+the+evolution+of+human+life+history)

Cutler GB Jr, Glenn M, Bush M, Hodgen GD, Graham CE, Loriaux DL 1978 Adrenarche: a survey of rodents, domestic animals, and primates. *Endocrinology* **103**(6):2112-2118. (https://www.ncbi.nlm.nih.gov/pubmed/?term=Adrenarche%3A+a+survey+of+rodents%2C+domestic+animals%2C+and+primates)

D'Amour DE, Hohmann G, Fruth B 2006 Evidence of leopard predation on bonobos (Pan paniscus). *Folia Primatol (Basel)* **77**(3):212-217. (https://www.ncbi.nlm.nih.gov/pubmed/?term=Evidence+of+leopard+predation+on+bonobos

Denis E, Pedentchouk N, Schouten S, Pagani M, Freeman KH 2017 Fire and ecosystem change in the Arctic across the Paleocene-Eocene Thermal Maximum. *Earth and Planetary Science Letters* **467**:149-156. (https://pennstate.pure.elsevier.com/en/publications/fire-and-ecosystem-change-in-the-arctic-across-the-paleoceneeocen)

Dunn RH, Rose KD, Rana RS, Kumor K, Sahni A, Smith T 2016 New euprimate postcrania from the early Eocene of Gujarat, India, and the strepsirrhine–haplorrhine divergence. *Journal of Human Evolution* **99**:25-51. (https://www.ncbi.nlm.nih.gov/pubmed/27650579)

Einaudi L, Courbiere B, Tassistro V, Prevot C, Sari-Minodier I, Orsiere T, Perrin J 2014 *In vivo* exposure to benzo(a)pyrene induces significant DNA damage in mouse oocytes and cumulus cells. *Human Reproduction* **29**(3):548-554. (https://www.ncbi.nlm.nih.gov/pubmed/?term=In+vivo+exposure+to+benzo(a)pyrene+induces+significant+DNA+damage+in+mouse+oocytes+and+cumulus)

Fowler PA, Childs AJ, Courant F, MacKenzie A, Rhind SM, Antignac JP, Le Bizec B, Fillis P, Evans F, Maheshwari A 2014 *In utero* exposure to cigarette smoke dysregulates human fetal ovarian developmental signaling. *Human Reproduction* **29**(7):1471-1489. (https://www.ncbi.nlm.nih.gov/pubmed/?term=In+utero+exposure+to+cigarette+smoke+dysregulates+human+fetal+ovarian+developmental+signaling)

Gassmann K, Abel J, Bothe H, Haarmann-Stemmann T, Merk HF, Quasthoff KN, Rockel TD, Schreiber T, Fritsche E 2010 Species-Specific Differential AhR Expression Protects Human Neural Progenitor Cells against Developmental Neurotoxicity of PAHs. *Environmental Health Perspectives* **118**(11): 1571–1577. (https://www.ncbi.nlm.nih.gov/pmc/articles/PMC2974695/)

Ge C, Ye J, Wang Q, Zhang C, Yang JM, Quian G 2012 Polycyclic aromatic hydrocarbons suppress meiosis in primordial germ cells via the AHR signaling pathway. *Toxicology Letters* **210**(3):285-292. (https://www.ncbi.nlm.nih.gov/pubmed/?term=Polycyclic+aromatic+hydrocarbons+suppress+meiosis+in+primordial+germ+cells+via+the+AHR+signaling+pathway)

Goodall J. The chimpanzees of Gombe: Patterns of behavior. Cambridge, MA: Belknap Press, 1986.

Goodman M, Porter CA, Czelusniak J, Page SL, Schneider H, Shoshani J, Gunnel G, Groves CP 1998 Toward a phylogenetic classification of Primates based on DNA evidence complemented by fossil evidence. *Molecular Phylogenetics and Evolution* **9**:585–598. (https://www.ncbi.nlm.nih.gov/pubmed/?term=Toward+a+phylogenetic+classification+of+Primates+based+on+DNA+evidence+complemented+by+fossil+evidence)

Haccard O, Dupré A, Liere P, Pianos A, Eychenne B, Jessus C, Ozon R 2012 Naturally occurring steroids in Xenopus oocyte during meiotic maturation. Unexpected presence and role of steroid sulfates. *Molecular and Cellular Endocrinology* **362**(1-2):110-119. (https://www.ncbi.nlm.nih.gov/pubmed/?term=Naturally+occurring+steroids+in+Xenopus+oocyte+during+meiotic+maturation.+Unexpected+presence+and+role+of+steroid+sulfates)

Heinrich U, Roller M, Pott F 1994 Estimation of a lifetime unit lung cancer risk for benzo(a)pyrene based on tumour rates in rats exposed to coal tar/pitch condensation aerosol. *Toxicology Letters* **72**(1-3):155-161. (https://www.ncbi.nlm.nih.gov/pubmed/?term=Estimation+of+a+lifetime+unit+lung+cancer+risk+for+benzo(a)pyrene+based+on+tumour+rates+in+rats+exposed+to+coal+tar%2Fpitch+condensation+a)

Hubbard TD, Murray IA, Bisson WH, Sullivan AP, Perry GH, Jablonski NG, Perdew GH 2016 Divergent Ah Receptor Ligand Selectivity during Hominin Evolution. *Molecular Biology and Evolution* **33**(10):2648–2658. (https://www.ncbi.nlm.nih.gov/pmc/articles/PMC5026259/)

Kee K, Flores M, Cedars MI, Reijo Pera RA 2010 Human primordial germ cell formation is diminished by exposure to environmental toxicants acting through the AHR signaling pathway. *Toxicological Sciences* **117**(1):218-224. (https://www.ncbi.nlm.nih.gov/pmc/articles/PMC2923286/)

Kim KH, Jahan SA, Kabir E, Brown RJ 2013 A review of airborne polycyclic aromatic hydrocarbons (PAHs) and their human health effects. *Environment International* **60**:71-80. (https://www.ncbi.nlm.nih.gov/pubmed/24013021)

Li Q, Li Y, Zhu L, Xing B, Chen B 2017 Dependence of Plant Uptake and Diffusion of Polycyclic Aromatic Hydrocarbons on the Leaf Surface Morphology and Micro-structures of Cuticular Waxes. *Scientific Reports* **7**:46235. (https://www.ncbi.nlm.nih.gov/pmc/articles/PMC5385540/)

Liu C, Ma Z, Xu S, Hou J, Hu Y, Yu Y, Liu R, Chen Z, Lu Y 2014 Activation of the germ-cell potential of human bone marrow-derived cells by a chemical carcinogen. *Scientific Reports* **4**:5564. (https://www.nature.com/articles/srep05564)

Lutz CT, Browne G, Petzold CR 1998 Methylcholanthrene causes increased thymocyte apoptosis. *Toxicology* **128**(2):151-167. (https://www.ncbi.nlm.nih.gov/pubmed/?term=Methylcholanthrene+causes+increased+thymocyte+apoptosis)

Mattison DR, Shiromizu K, Nightingale MS 1983 Oocyte destruction by polycyclic aromatic hydrocarbons. *American Journal of Industrial Medicine* **4**(1-2):191-202. (https://www.ncbi.nlm.nih.gov/pubmed/6301272)

Miller ER, Gunnell GF, Martin RD 2005 Deep time and the search for anthropoid origins. *Yearbook of Physical Anthropology* **48**:60-95. (https://www.ncbi.nlm.nih.gov/pubmed/?term=Deep+time+and+the+search+for+anthropoid+origins)

O'Brien JM, Beal MA, Yauk CL, Marchetti F 2016 Next generation sequencing of benzo(a)pyrene-induced lacZ mutants identifies a germ cell-specific mutation spectrum. *Scientific Reports* **6**:36743. (https://www.nature.com/articles/srep36743)

Liu C, Ma Z, Xu S, Hou J, Hu Y, Yu Y, Liu R, Chen Z, Lu Y 2014 Activation of the germ-cell potential of human bone marrow-derived cells by a chemical carcinogen. *Scientific Reports* **4**:5564. (https://www.nature.com/articles/srep05564)

Lutz CT, Browne G, Petzold CR 1998 Methylcholanthrene causes increased thymocyte apoptosis. *Toxicology* **128**(2):151-167. (https://www.ncbi.nlm.nih.gov/pubmed/?term=Methylcholanthrene+causes+increased+thymocyte+apoptosis)

Ni X, Gebo DL, Dagosto M, Meng J, Tafforeau P, Flynn JJ, Beard KC 2013 The oldest known primate skeleton and early haplorrhine evolution. *Nature* **498**:60-64. (https://www.nature.com/articles/nature12200)

Nyce, Jonathan. Species-specificity of an adrenal androgen-mediated kill switch that is triggered by the inactivation of the p53 tumor suppressor. *Translational Medicine Reports*, 2017, **1**:6773. (http://www.pagepressjournals.org/index.php/tmr/article/view/6773)

Parker CH, Keefe ER, Herzog NM, O'Connell JF, Hawkes K 2016 The pyrophilic primate hypothesis. *Evolutionary Anthropology* **25**(2):54-63. (https://www.ncbi.nlm.nih.gov/pubmed/?term=The+pyrophilic+primate)

Perelman P, Johnson WE, Roos C, Seuanez HN, Horvath JE, Moreira MAM, Kessing B, Pontius J, Roelke M, Rumpler Y, *et al.* 2011 A Molecular Phylogeny of Living Primates. *PLoS Genet* **7**(3): e1001342. (https://www.ncbi.nlm.nih.gov/pmc/articles/PMC3060065/)

Perez-Fernandez R, Facchinetti F, Beiras A, Lima L, Gaudiero GJ, Genazzani AR, Devesa J 1987 Morphological and functional stimulation of adrenal reticularis zone by dopaminergic blockade in dogs. *Journal of Steroid Biochemistry* **28**(5):465-470. (https://www.ncbi.nlm.nih.gov/pubmed/?term=Morphological+and+functional+stimulation+of+adrenal+reticularis+zone+by+dopaminergic+blockade+in+dogs)

Perret M, Aujard F 2005 Aging and season affect plasma dehydroepiandrosterone sulfate (DHEA-S) levels in a primate. *Experimental Gerontology* **40**(7):582-587. (https://www.ncbi.nlm.nih.gov/pubmed/?term=Aging+and+season+affect+plasma+dehydroepiandrosterone+sulfate+(DHEA-S)+levels+in+a+primate)

Phillips DH 1999 Polycyclic aromatic hydrocarbons in the diet. *Mutation Research* **443**(1-2):139-147. (https://www.ncbi.nlm.nih.gov/pubmed/10415437)

Pruetz JD, LaDuke TC 2010 Reaction to fire by savanna chimpanzees (*Pan troglodytes*) at Fongoli, Senegal: Conceptualization of "fire behavior" and the case for a chimpanzee model. *American Journal of Physical Anthropology* **141**(4):646-650. (https://www.ncbi.nlm.nih.gov/pubmed/?term=Reaction+to+fire+by+savanna+chimpanzees+(Pan+troglodytes)+at+Fongoli%2C+Senegal%3A+Conceptualization+of+%E2%80%9Cfire+behavior%E2%80%9D+and+the+case+for+a+chimpanzee+model)

Robson BE, Collinson ME, Riegel W, Wilde V, Scott AC, Pancoast RD 2014 A record of fire through the Early Eocene. *Rend Online Societa Geologica Italiana* **31**:187-188. (https://www.researchgate.net/publication/271847726_A_record_of_fire_through_the_Early_Eocene)

Schiebinger RJ, Albertson BD, Barnes KM, Cutler GB Jr, Loriaux DL 1981 Developmental changes in rabbit and dog adrenal function: a possible homologue of adrenarche in the dog. *American Journal of Physiology* **240**(6):E694-9. (https://www.ncbi.nlm.nih.gov/pubmed/6264794)

Selkin PA, Stromberg CAE, Dunn R, Kohn MJ, Carlini AA, Davies-Vollum KS, Madden RH 2015 Climate, dust, and fire across the Eocene-Oligocene Transition, Patagonia. *Geology* **43**(7):567-570. (https://pubs.geoscienceworld.org/gsa/geology/article-abstract/43/7/567/131882/climate-dust-and-fire-across-the-eocene-oligocene?redirectedFrom=fulltext)

Suzuki M, Wright LS, Marwah P, Lardy HA, Svendsen CN 2004 Mitotic and neurogenic effects of Dehydroepiandrosterone (DHEA) on human neural stem cell cultures derived from the fetal cortex. *Proceedings of the National Academy of Sciences (USA)* **101**(9):3202-3207. (http://www.pnas.org/content/101/9/3202.long)

Thomas DJ, Zachos JC, Bralower TJ, Thomas E, Bohaty S 2002 Warming the fuel for the fire: Evidence for the thermal dissociation of methane hydrate during the Paleocene-Eocene Thermal Maximum. *Geology* **30**(12):1067-1070. (https://pubs.geoscienceworld.org/gsa/geology/article-abstract/30/12/1067/192270/warming-the-fuel-for-the-fire-evidence-for-the?redirectedFrom=PDF)

Wang Y, He J, Wang S, Luo C, Yin H, Zhang G 2017 Characterization and risk assessment of polycyclic aromatic hydrocarbons (PAHs) in soils and plants around e-waste dismantling sites in southern China. *Environmental Science Pollution Research International* **439**:187-193. (https://www.ncbi.nlm.nih.gov/pubmed/23063924)

Zhang M, Miao Y, Chen Q, Cai M, Dong W, Dai X, Lu Y, Zhou C, Xiong B 2018 BaP exposure causes oocyte meiotic arrest and fertilization failure to weaken female fertility. *FASEB J* **32**(1):342-352. (https://www.ncbi.nlm.nih.gov/pubmed/?term=BaP+exposure+causes+oocyte+meiotic+arrest+and+fertilization+failure+to+weaken+female+fertility)
