# Supplementary material for: Detection of a novel, primate-specific ‘kill switch’ tumor suppression mechanism that may fundamentally control cancer risk in humans: an unexpected twist in the basic biology of TP53
Source: Endocr Relat Cancer. 2018 Jun 25;25(11):R497–517. doi: 10.1530/ERC-18-0241 (PMC6106910; doi:10.1530/ERC-18-0241)
Supplement: Supplementary Section 2 [file erc-25-R497-s002.pdf]

Supplemental Section 2

Species (Accession Number):

Rattus norvegicus (013098.2)

Mus musculus (U91573)

Mongolian Gerbil (XM_021641187)

Prairie Vole (NW_004949142.1)

Deer Mouse (XM_006992939.2)

Chinese Tree Shrew (XM_006156078.2)

Naked Mole Rat (JH169284)

Dammaraland Mole Rat (KN122754)

Blind Mole Rat (NW_008340203)

Kangaroo Rat (NW_012267223.1)

Degu (NW_004524632.1)

Beaver (NW_017870580.1)

Guinea Pig (XM_003467130)

Elephant (NW_003573451.1)
Rabbit (XM_002719415)

Egyptian Fruit Bat (XM_016150937)

Killer Whale (XM_004282844)

Beluga Whale (XM_022558106.1)

Bottlenose Dolphin (XM_004330514.2)

Nine-banded Armadillo (XM_004484581)

Cattle (XM_005220740)

Water Buffalo (NW_005784969)

Southern White Rhino (XM_004434415.2)

Human (NG_011808)

Chimpanzee (XM_016931752)

Sooty Mangabey (XM_012046770.1)

Rhesus Monkey (NW_014805577.1)

Green Monkey (XM_008012558)

Black Snub-nosed Monkey (XM_017867665)

Tufted White Eared Marmoset (XM_008996672)

Tarsier (NW_007253994.1)

Gray Mouse Lemur (XM_012743277)

Sunda Flying Lemur (NW_007726844.1)

Canine (AF295565)
