# Supplementary material for: Detection of a novel, primate-specific ‘kill switch’ tumor suppression mechanism that may fundamentally control cancer risk in humans: an unexpected twist in the basic biology of TP53
Source: Endocr Relat Cancer. 2018 Jun 25;25(11):R497–517. doi: 10.1530/ERC-18-0241 (PMC6106910; doi:10.1530/ERC-18-0241)
Supplement: Supplementary Section 3 [file erc-25-R497-s003.pdf]

Supplemental Section 3

Extended discussion: Tumor suppression in the elephant and the Naked Mole Rat

The elephant and the Naked Mole Rat (NMR) are two species that enjoy long life spans and an extremely low lifetime risk of cancer (Abegglen *et al*., 2015; Miyawaki *et al*., 2016). Each of these species has a large complement of processed pseudogenes (retropseudogenes) of one or the other of arguably the two most important tumor suppressors in cancer. The elephant has 19 retropseudogenes for p53 (Abegglen *et al*., *ibid*.; Sulak *et al*., 2016), collectively called p53p; and the NMR has 17 retropseudogenes for PTEN (Tang *et al*., 2016), collectively called PTENp. This struck us as deeply interesting because both p53 and PTEN are direct inhibitors of G6PD, blocking the formation of active G6PD dimers by directly binding to the enzyme monomer (Jiang *et al*., 2011; Hong *et al*, 2014). Thus, despite the fact that the elephant and the NMR are on opposite ends of the spectrum of mammalian body weight, their trajectories through spacetime may have adopted inhibition of G6PD *via* parallel species-specific mechanisms of tumor suppression. This could mean that all currently identified mechanisms of species-specific tumor suppression might have the same ultimate target, G6PD— primates by conversion of circulating DHEAS to DHEA, an irreversible uncompetitive inhibitor of G6PD; and the elephant and the NMR by incorporation of, respectively, multiple copies of p53p and PTENp into their genomes. If this is true, it suggests that inhibition of G6PD is a common mechanism to extinguish cells that have experienced potentially tumorigenic mutations in p53, or possibly also PTEN.

Both p53p in the elephant, and PTENp in the NMR are expressed and produce RNA transcripts, albeit from adopted promoters, not the p53 or PTEN gene promoters (Abegglen *et al., ibid*; Sulak *et al, ibid*.; Tang *et al, ibid*.). It is of particular interest that several of the elephant p53ps have also been demonstrated to be translated into protein (Abegglen, *ibid*.). How might p53p and PTENp expressed retropseudogenes participate in a kill switch targeting G6PD? PTENp retropseudogenes are established as tumor suppressors, and their loss has been demonstrated in a variety of human cancers (Grander and Johnsson, 2016; Gao *et al*., 2016). Poliseno and colleagues (2010) have provided evidence that PTENp pseudogene transcripts function as competitive endogenous RNAs (ceRNAs) that act as a sink for microRNAs (miRNAs) that would

otherwise inhibit the translation of PTEN transcripts. Cells are ultrasensitive to even slight variations in PTEN (Alimonti *et al*., 2010). Protection from PTEN translation inhibition afforded by the 17 PTENp retropseudogenes acting as ceRNA in the NMR may enable PTEN protein levels to rise rapidly and effect a super PTEN phenotype, directly inhibiting G6PD and thereby inducing ROS-mediated cell death. But we believe that the NMR has evolved an even more complex species-specific response to counter neoplastic transformation.

Some specifics and speculations on the Naked Mole Rat's trajectory through spacetime

As discussed in the main article, *all* enabling elements of a species' forward movement through spacetime are under simultaneous and integrated selection, such that species-specific traits that evolved to exploit a particular environment can be recruited to augment canonical mechanisms of tumor suppression if they possess properties relevant to this goal. Two primary features of the subterranean environment of the NMR are anoxia and aridity. NMRs appear to have solved the oxygen deprivation problem by evolving fructose-driven glycolysis, thereby avoiding feedback inhibition of glycolysis *via* phosphofructokinase (Park *et al*., 2017). This enables them to survive long periods of relative anoxia. We propose that, additionally, NMRs utilize the polyol pathway to generate supplementary fructose from glucose, thereby fueling fructose-driven glycolysis to the levels required to dwell in the anoxic tunnelways and burrows that constitute their environment.

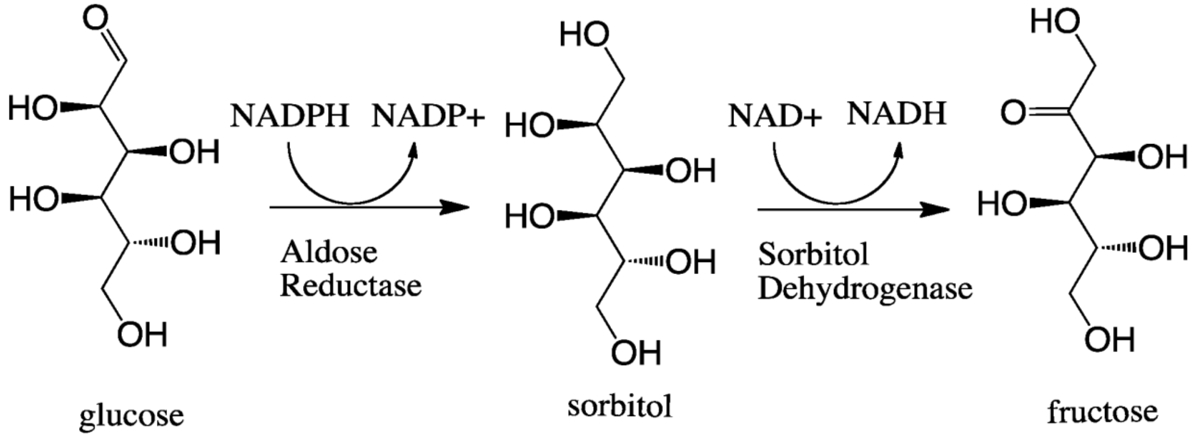

Figure 3-1. The Polyol Pathway can produce the additional fructose that the NMR may require to exploit its subterranean environment, but at the cost of expenditure of NADPH that would ordinarily be required to reduce ROS to survivable levels.

As can be seen in Figure 3-1, the strategy of employing the polyol pathway to fuel fructose-driven glycolysis would come at the cost of Aldose Reductase acting as a sink for intracellular NADPH. As a general rule, NADPH is required to maintain ROS at levels that are survivable by the cell. The polyol pathway is well known to be a source of oxidative stress (Panigrahy *et al*., 2017), and NMR cells have been found to have high levels of oxidative damage (Andziak *et al*., 2006). Additional evidence for use of the polyol pathway by NMR cells, which would be at the expense of NADPH-requiring enzymes, comes from the discovery that the NMR has abandoned Glutathione Peroxidase (GPX) (Kasaikina *et al*., 2011), a critical component of the NADPH-dependent anti-oxidant system of other species. The NMR may thus have abandoned GPX because its species-specific approach to the exploitation of its environment required that substantial NADPH be directed toward operation of the polyol pathway, leaving insufficient NADPH remaining to operate GPX. If this is true, then an alternative, species-specific mechanism to modulate ROS levels must also have evolved in the NMR. We believe that such mechanism involves a species-specific, extremely high molecular weight hyaluronic acid (HMW HA) that Tian and colleagues have recently discovered in this species (Tian *et al*., 2013). In keeping with the well-known role of HA in formation of the extracellular matrix, these authors have proposed that extracellular properties of this HMW HA unique to the NMR may be responsible for the extreme cancer resistance of this species. They have provided evidence that NMR fibroblasts demonstrate early contact inhibition *in vitro* and have shown that this is a p53- and RB-dependent phenomenon (Seluanoy *et al*., 2009). But there is also evidence that *intracellular* HA plays an important role in the physiology of the cell, and both intracellular and even intranuclear HA have been detected (Hascall *et al*., 2004; Adamia *et al*., 2005; Harada and Takahashi, 2007). HA is also well known to be a potent anti-oxidant (Sudha and Rose, 2014), and this fact— coupled with the discovery of 17 PTENp in the NMR genome— encourages us to suggest that the HMW HA of the NMR participates in a kill switch tumor suppression mechanism that is triggered by inactivation of p53, and which ultimately targets G6PD, just as the primate-specific adrenal androgen-mediated kill switch does. The potential role of HMW HA as a component of the NMR's tumor suppression mechanism would nicely illustrate the concept of coordinated selection for multiple tasks, as HA is one of Nature's best moisturizing agents

(Salwowska *et al*., 2016). The tissue hydrating properties of HMW HA may therefore have enabled the NMR to exploit the arid subterranean environment as its habitat.

According to the scenario that we here present, the potent antioxidant properties of HMW HA may maintain a relatively pristine nuclear environment for the NMR genome, and may play the major role in control of intracellular ROS levels— a role played by NADPH in most other species. When p53 inactivation does occur, de-repressed NFkB initiates a Hyaluronidase-driven degradation of HMW HA (Lokeshwar *et al*., 2008). Low Molecular Weight (LMW) HA is inflammatory (Ghosh *et al*., 2015) and activates PTEN (Ghatak *et al*., 2002). As previously noted, PTEN is a direct inhibitor of G6PD (Hong *et al*., 2014), and G6PD inhibition raises ROS/NOS levels (Stanton, 2012). ROS/NOS degrade HMW HA into LMW HA (Soltes *et al*., 2006). As ROS/NOS levels increase, NFkB activity is further stimulated (Morgan and Liu, 2011), and the degradation of the HMW HA accelerates. PTEN also inhibits Hyaluronan Synthetase (Geng *et al*., 2016), which prevents the pool of intracellular HMW HA from being refilled. The increasing levels of low molecular weight HA drive PTEN inhibition of G6PD, raising ROS/NOS levels still further by depletion of both NADPH *and* HMW HA. PTENp pseudogenes are also induced and act as ceRNA to further increase PTEN activity (Grander and Johnsson, 2016; Gao *et al*., 2016). De-repression of G6PC by the loss of p53 depletes intracellular G6P by accelerating its conversion into glucose, and then into fructose *via* aldose reductase (AR), the first step in the polyol pathway. AR activity acts as a sink for NADPH, thereby contributing to its depletion, with the buildup of intracellular sorbitol as NFkB drives glucose use away from glycolysis (Mauro *et al*., 2011). Sorbitol itself induces hyperosmotic stress and apoptosis (Olivera Santa-Catalina *et al*., 2017).

According to the hypothesis presented here, HMW HA and NADPH are simultaneously withdrawn in a species-specific manner in the p53-inactivated NMR cell, leading to ROS-mediated cell death. In the species-specific trajectory of the NMR through spacetime, HMW HA thus evolved to enable exploitation of an arid subterranean environment, and was simultaneously incorporated into a kill switch mechanism paralleling that which we have detected in primates. As we shall see below, this targeting of G6PD by kill switch tumor suppression mechanisms may continue in the elephant.

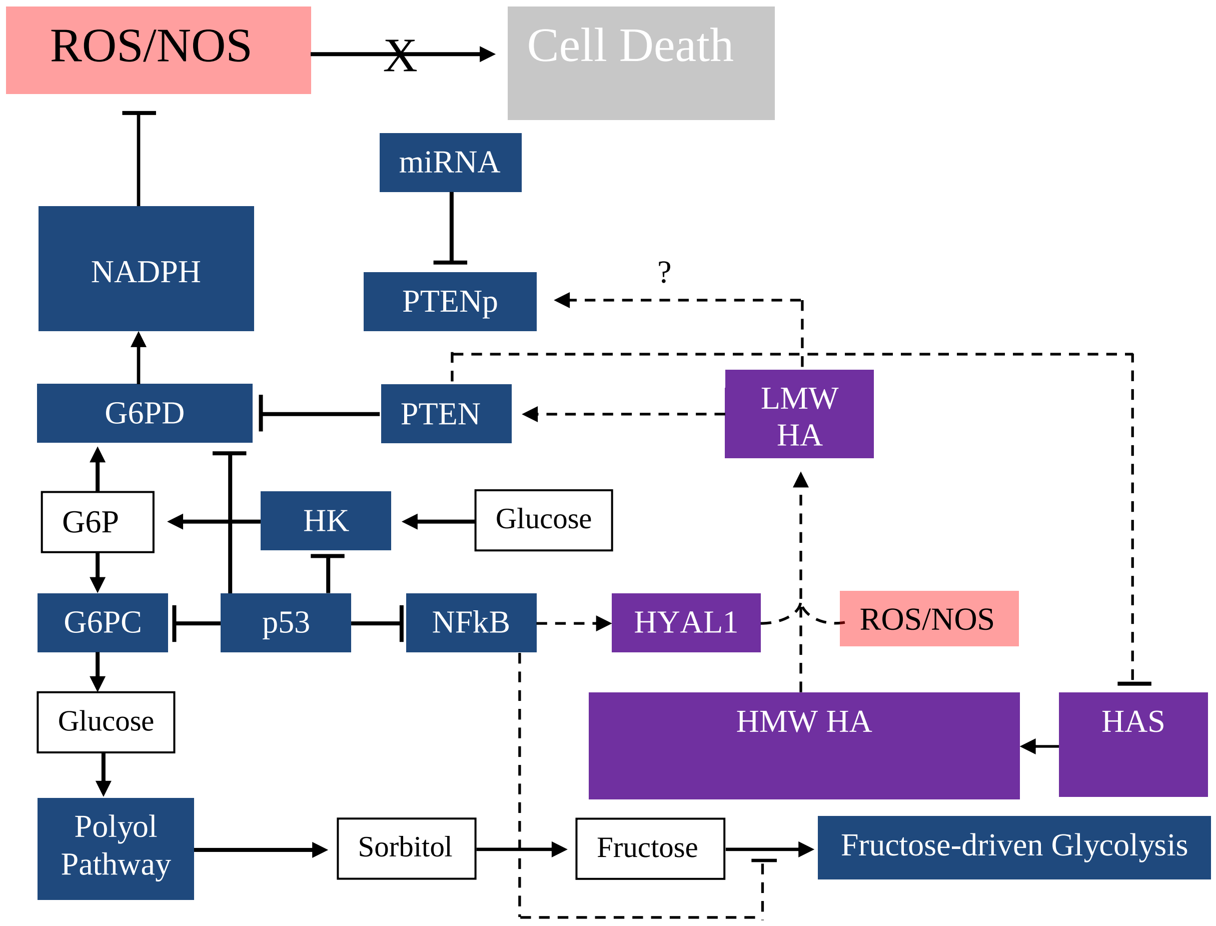

Supplemental Section 3 Figure 1 A. Proposed mechanism of species-specific kill switch employing High Molecular Weight (HMW) Hyaluronic Acid (HA) in the Naked Mole Rat (NMR). Among its myriad duties, p53 regulates G6PD (Jiang *et al.*, 2011), NFkB (Carroll *et al.*, 2016; Ghose *et al.*, 2011; Webster and Perkins, 1999), and G6PC (Zhang *et al.*, 2014). Under normal hypoxic conditions in the NMR, G6PC is active, metabolizing some fraction of G6P to glucose, which can then be further metabolized to fructose *via* the polyol pathway. We propose that the kill switch trigger in the NMR is centered on p53 inhibition of NFkB just as it is in the primate. However, instead of Steroid Sulfatase and circulating DHEAS as the firing mechanism, as in primates, the NMR kill switch mechanism consists of Hyaluronidase I (HYAL1), an initial large pool of intracellular HMW HA, and the potential for PTEN to cut off resupply of HMW HA by inhibiting Hyaluronan Synthase (Geng *et al.*, 2016). The model requires the presence of PTENp retropseudogenes acting as ceRNA to absorb the microRNAs that would otherwise inhibit PTEN translation (Tang *et al.*, 2016). PTENp expression might be stimulated by LMW HA, as PTEN is known to be (Ghatak *et al.*, *ibid*.)

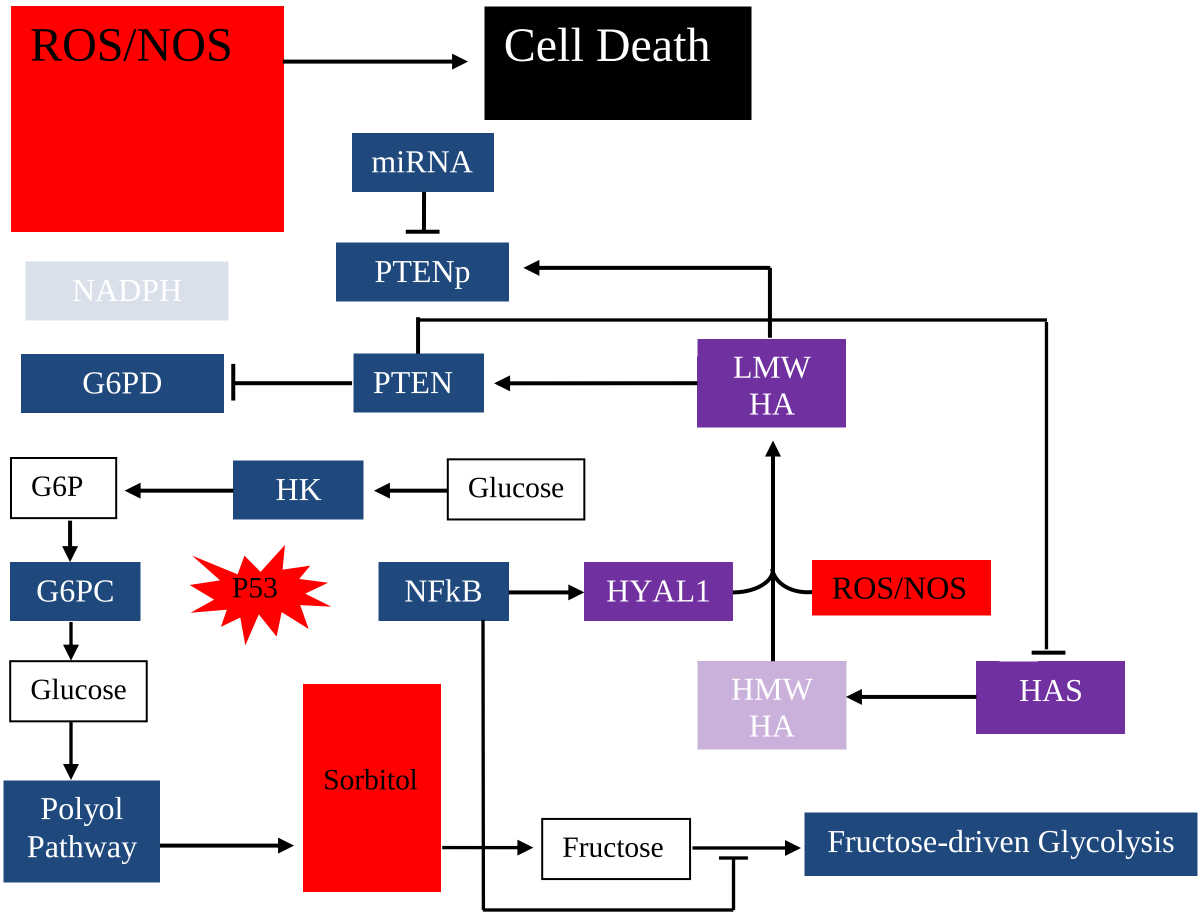

Supplemental Section 3 Figure 1 B. Proposed mechanism of species-specific kill switch employing High Molecular Weight (HMW) Hyaluronic Acid (HA) in the Naked Mole Rat (NMR), continued. In the event of p53 inactivation in a somatic cell of the NMR, a kill switch mechanism is triggered in which HYAL1 activity degrades a standing large anti-oxidant pool of HMW HA. LMW HA stimulates PTEN (and possibly PTENp), which prevents replenishment of HMW HA by inhibition of HAS. NADPH pools are eliminated by direct inhibition of G6PD by PTEN, and by activation of the polyol pathway which acts as a sink for NADPH. Because NFkB drives glucose utilization away from glycolysis and toward oxidative phosphorylation, sorbitol may also reach levels associated with apoptosis. Abbreviations: G6PD, Glucose-6-phosphate Dehydrogenase. ROS, Reactive Oxygen Species. NOS, Reactive Nitrogen Species. PTEN, Phosphatase and Tensin Homologue. PTENp, pseudigene of PTEN capable of RNA transcript expression. LMW HA, Low Molecular Weight Hyaluronic Acid. HMW HA, High Molecular Weight Hyaluronic Acid. HK, Hexokinase. G6P, Glucose-6-phosphate, the substrate of G6PD. G6PC, Glucose-6-phosphatase. HYAL1, Hyaluronidase 1. HAS, Hyaluronic Acid Synthetase. NFkB, Nuclear Factor Kappa B.

Some specifics and speculations on the Naked Mole Rat's trajectory through spacetime

As noted above, elephants appear to suppress cancer by amplifying the number of p53p retropseudogenes in their genomes (Abegglen *et al*., *ibid*.; Sulak *et al*., 2016). While these p53p retropseudogenes may function in a manner parallel to PTENp retropseudogenes, acting as ceRNA to absorb miRNA that would otherwise prevent translation of p53, the fact that at least some of the elephant p53p retropseudogenes appear to be translated into protein suggest the possibility that they may alternatively (or additionally) act as direct inhibitors of G6PD, blocking the formation of active G6PD dimers, as p53 itself is capable of doing. In such case, inactivation of one of the p53 alleles could trigger a storm of G6PD inhibition by the p53p retrospeudogene corps. Whatever the specific mechanism of p53p retropseudogene action to restrain tumorigenesis in the elephant turns out to be, the point to be made here is that in this species, in the Naked Mole Rat, and in primates, there is a connection: p53, PTEN and DHEA are each known inhibitors of G6PD, an enzyme that is essential to the control of intracellular ROS. This could be coincidence, or it could mean that G6PD is the ultimate target of kill switch tumor suppression systems in general, perhaps including additional ones yet to be uncovered.

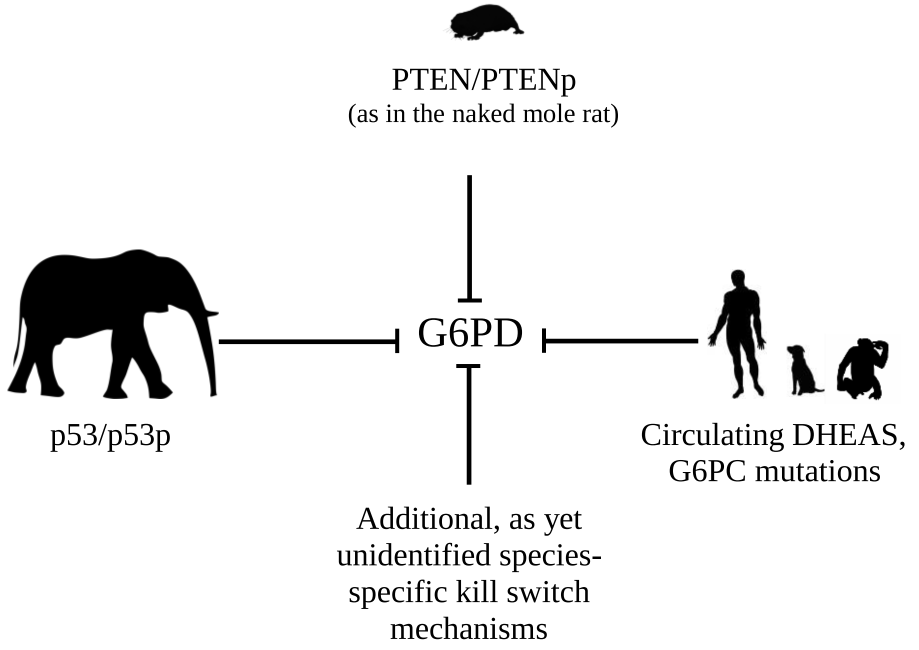

References for Supplemental Section 3

Abegglen LM, Caulin AF, Chan A, Lee K, Robinson R, Campbell MS, Kiso WK, Schmitt DL, Waddell PJ, Bhaskara S, *et al*. 2015 Potential Mechanisms for Cancer Resistance in Elephants and Comparative Cellular Response to DNA Damage in Humans. *Journal of the American Medical Association* **314**(17):1850-1860. (https://jamanetwork.com/journals/jama/fullarticle/2456041)

Adamia S, Maxwell CA, Pilarski LM 2005 Hyaluronan and hyaluronan synthases: potential therapeutic targets in cancer. *Current Drug Targets in Cardiovascular and Haematological Disorders* **5**(1):3-14. (http://www.eurekaselect.com/90374/article)

Alimonti A, Carracedo A, Clohessy JG, Trotman LC 2010 Subtle variations in PTEN dose determine cancer susceptibility. *Nature Genetics* **42**(5): 454–458. (https://www.ncbi.nlm.nih.gov/pmc/articles/PMC3118559/)

Andziak B, O'Connor TP, Qi W, DeWaal EM, Pierce A, Chaudhuri A, Van Remmen H, Buffenstein R 2006 High oxidative damage levels in the longest-living rodent, the naked mole-rat. *Aging Cell* **5**(6):463-471 (https://www.ncbi.nlm.nih.gov/pubmed/17054663)

Carroll BL, Pulkoski-Gross ML, Hannun YA, Obeid LM 2016 CHK1 regulates NFkB signaling upon DNA damage in p53- deficient cells and associated tumor-derived microvesicles. *Oncotarget* **7**(14): 18159–18170. (https://www.ncbi.nlm.nih.gov/pmc/articles/PMC4951279/)

Gao L, Ren W, Zhang L, Li S, Kong X, Zhang H, Dong J, Cai G, Jin C, Zheng D *et al*. 2016 PTENp1, a natural sponge of miR-21, mediates PTEN expression to inhibit the proliferation of oral squamous cell carcinoma.

Molecular Carcinogenesis **56**(4):1322-1334. (https://www.ncbi.nlm.nih.gov/pubmed/27862321)

Ghatak S, Misra S, Toole BP 2002 Hyaluronan Oligosaccharides Inhibit Anchorage-independent Growth of Tumor Cells by Suppressing the Phosphoinositide 3-Kinase/Akt Cell Survival Pathway. *The Journal of Biological Chemistry* **377**:38013-38020. http://www.jbc.org/content/277/41/38013.long

Geng J, Huang X, Li Y, Xu X, Li S, Jiang D, Liu Z, Dai H 2016 Phosphatase and tensin homolog deleted on chromosome 10 contributes to phenotype transformation of fibroblasts in idiopathic pulmonary fibrosis via multiple pathways. *Experimental Biology and Medicine* **241**(2):157-165. (http://journals.sagepub.com/doi/abs/10.1177/1535370215600100?url_ver=Z39.88-2003&rfr_id=ori:rid:crossref.org&rfr_dat=cr_pub%3dpubmed)

Ghose J, Sinha M, Das E, R. Jana NR, Bhattacharyya NP 2011 Regulation of miR-146a by RelA/NFkB and p53 in STHdh^{Q111}/Hdh^{Q111} Cells, a Cell Model of Huntington's Disease. *PLoS One* **6**(8): e23837. (https://www.ncbi.nlm.nih.gov/pmc/articles/PMC3162608/)

Ghosh S, Hoselton SA, Dorsam GP, Schuh JM 2015 Hyaluronan fragments as mediators of inflammation in allergic pulmonary disease. *Immunobiology* **220**(5):575-588. (https://www.ncbi.nlm.nih.gov/pmc/articles/PMC4393768/)

Grander D and Johnsson P 2016 Pseudogene-expressed RNAs: Emerging roles in gene regulation and disease. *Current Topics in Microbiology and Immunology* **394**:111-126. (https://www.ncbi.nlm.nih.gov/pubmed/25982975)

Grempler R, Kienitz A, Werner T, Meyer M, Barthel A, Ailett F, Sutherland C, Walther R, Schmoll D 2004 Tumour necrosis factor alpha decreases glucose-6-phosphatase gene expression by activation of nuclear factor kappaB. *Biochemical Journal* **382**(Pt 2):471-9. (https://www.ncbi.nlm.nih.gov/pmc/articles/PMC1133803/)

Hascall VC, Majors AK, De La Motte CA, Evanko SP, Wang A, Drazba JA, Wight TN 2004 Intracellular hyaluronan: a new frontier for inflammation? *Biochimica et Biophysica Acta* **1673**(1-2):3-12. (https://www.ncbi.nlm.nih.gov/pubmed/15238245)

Harada H, Takahashi M 2007 CD44-dependent intracellular and extracellular catabolism of hyaluronidase-1 and -2. *Journal of Biological Chemistry* **282**: 5597-5607. (http://www.jbc.org/content/282/8/5597.long)

Hong X, Song R, Song H, Zheng T, Wang J, Liang Y, Qi S, Lu Z, Song X, Jiang H, *et al*. 2014 PTEN antagonizes Tcl1/hnRNPK-mediated G6PD pre-mRNA splicing which contributes to hepatocarcinogenesis. *Gut* **63**:1635-1647. (https://www.ncbi.nlm.nih.gov/pubmed/?term=PTEN+antagonises+Tcl1%2FhnRNPK-mediated+G6PD+pre-mRNA+splicing+which+contributes+to+hepatocarcinogenesis)

Jiang P, Du W, Wang X, Mancuso A, Gao X 2011 p53 regulates biosynthesis through direct inactivation of glucose-6-phosphate dehydrogenase. *Nature Cell Biology* **13**(3):310- 316. (https://www.ncbi.nlm.nih.gov/pmc/articles/PMC3110666/)

Kasaikina MV, Lobanov AV, Malinouski MY, Lee BC, Seravalli J, Fomenko DE, Turanov AA, Finney L, Vogt S, Park TJ, *et al*. 2011 Reduced Utilization of Selenium by Naked Mole Rats Due to a Specific Defect in GPx1 Expression. Journal of Biological Chemistry **286**(19):17005–17014 (https://www.ncbi.nlm.nih.gov/pmc/articles/PMC3089545/)

Lokeshwar VB, Gomez P, Kramer M, Knapp J, McCornack MA, Lopez LE, Fregien N, Dhir N, Scherer S, Klumpp DJ, *et al*. 2008 Epigenetic regulation of HYAL-1 hyaluronidase expression. identification of HYAL-1 promoter. *Journal of Biological Chemistry* **283**(43):29215-29227. (http://www.jbc.org/content/283/43/29215.long)

Majumdar M, Meenakshi J, Goswami SK, Datta K 2002 Hyaluronan binding protein 1 (HABP1)/C1QBP/p32 is an endogenous substrate for MAP kinase and is translocated to the nucleus upon mitogenic stimulation. *Biochemical and Biophysical Research Communications* **291**(4):829-37. (https://www.ncbi.nlm.nih.gov/pubmed/?term=Hyaluronan+binding+protein+1+(HABP1)%2FC1QBP%2Fp32+is+an+endogenous+substrate+for+MAP+kinase+and+is+translocated+to+the+nucleus+upon+mitogenic+stimulation)

Mauro C, Leow SC, Anso E, Rocha S, Thotakura AK, Tornatore L., Morettin M, De Smaele E, Beg AA, Tergaonakar V *et al*. 2011 NF-kB controls energy homeostasis and metabolic adaptation by upregulating mitochondrial respiration. *Nature Cell Biology* **13**(10:1272-1279. (https://www.ncbi.nlm.nih.gov/pmc/articles/PMC3462316/)

Miyawaki S, Kawamura Y, Oiwa Y, Shimizu A, Hachiya T, Bono H, Koya I, Okada Y, Kimura T, Tsuchiya Y, *et al*. 2016 Tumour resistance in induced pluripotent stem cells derived from naked mole-

rats. *Nature Communications* **7**:11471. (https://www.ncbi.nlm.nih.gov/pmc/articles/PMC4866046/)

Olivera Santa-Catalina M, Caballero Bermejo M, Argent R, Centeno F, Lorenzo MJ 2017 JNK signaling pathway regulates sorbitol-induced Tau proteolysis and apoptosis in SH-SY5Y cells by targeting caspase-3. *Archives of Biochemistry and Biophysics* **636**:42-49. (https://www.ncbi.nlm.nih.gov/pubmed/29126968)

Panigrahy SK, Bhatt R, Kumar A (2017) Reactive oxygen species: sources, consequences and targeted therapy in type 2 diabetes. *Journal of Drug Targeting* **25**(2):93-101. (https://www.ncbi.nlm.nih.gov/pubmed/?term=Reactive+oxygen+species%3A+sources%2C+consequences+and+targeted+therapy+in+type+2+diabetes)

Park TJ, Reznick J, Peterson BL, Blass G, Omerbašić D, Bennett NC, Kuich PHJL, Zasada C, Browe BM, Hamann W, *et al*. (2017) Fructose-driven glycolysis supports anoxia resistance in the naked mole-rat. *Science* **356**(6335):307-311. (https://www.ncbi.nlm.nih.gov/pubmed/?term=Fructose-driven+glycolysis+supports+anoxia+resistance+in+the+naked+mole-rat)

Poliseno L, Salmena L, Zhang J, Carver B, Haveman WJ, Pandolfi PP 2010 A coding-independent function of gene and pseudogene mRNAs regulates tumour biology. *Nature* **465**(7301):1033–1038]. (https://www.ncbi.nlm.nih.gov/pmc/articles/PMC3206313/)

Salwowska NM, Bebenek KA, Żądło DA, Wcisło-Dziadecka DL 2016 Physiochemical properties and application of hyaluronic acid: a systematic review. *Journal of Cosmetic Dermatology* **15**(4):520-526.
(https://www.ncbi.nlm.nih.gov/pubmed/?term=Physiochemical+properties+and+application+of+hyal

uronic+acid%3A+a+systematic+review)

Seluanov A, Hine C, Azpurua J, Feigenson M, Bozzella M, Mao Z, Catania KC, Gorbunova V 2009 Hypersensitivity to contact inhibition provides a clue to cancer resistance of naked mole-rat. Proceedings of the *National Academy of Sciences USA* **106**:19207–19208. (https://www.ncbi.nlm.nih.gov/pmc/articles/PMC2780760/)

Soltés L, Mendichi R, Kogan G, Schiller J, Stankovska M, Arnhold J 2006 Degradative action of reactive oxygen species on hyaluronan. *Biomacromolecules* **7**(3):659-68. (https://www.ncbi.nlm.nih.gov/pubmed/16529395)

Stanton RC 2012 Glucose-6-phosphate Dehydrogenase, NADPH, and cell survival. *IUBMB Life* **54**(5):362-369. (https://www.ncbi.nlm.nih.gov/pmc/articles/PMC3325335/)

Sudha PN, Rose MH 2014 Beneficial effects of hyaluronic acid. *Advances in Food and Nutrition Research* **72**:137-176. (https://www.ncbi.nlm.nih.gov/pubmed/?term=sudha+roseBeneficial+effects+of+hyaluronic+acid)

Sulak M, Fong L, Mika K, Chigurupati S, Yon L, Mongan NP, Emes RD, Lynch VJ 2016 *TP53* copy number expansion is associated with the evolution of increased body size and an enhanced DNA damage response in elephants. *eLife* 5:e11994 (https://www.ncbi.nlm.nih.gov/pmc/articles/PMC5061548/)

Tang J, Ning R, Zeng B, Li Y 2016 Molecular Evolution of PTEN Pseudogenes in Mammals. *PLoS One* **11**(12):e0167851. (https://www.ncbi.nlm.nih.gov/pmc/articles/PMC5148010/)

Tian X, Azpurua J, Hine C, Vaidya A, Myakishev-Rempel M, Ablaeva J, Mao Z, Nevo E, Gorbunova V, Seluanov A 2013 High molecular weight hyaluronan mediates the cancer resistance of the naked mole-rat. *Nature* **499**(7458): 346–349. (https://www.ncbi.nlm.nih.gov/pmc/articles/PMC3720720/)

Webster GA, Perkins ND 1999 Transcriptional Cross Talk between NF-kB and p53. *Molecular and Cellular Biology* **19**(5): 3485–3495. (https://www.ncbi.nlm.nih.gov/pmc/articles/PMC84141/)

Zhang P, Tu B, Wang H, Cao Z, Tang M, Zhang C, Gu B, Li Z, Wang L, Yang Y, *et al.* 2014 Tumor suppressor p53 cooperates with SIRT6 to regulate gluconeogenesis by promoting FoxO1 nuclear exclusion. *Proceedings of the National Academy of Sciences U S A* **111**(29):10684-10689. (https://www.ncbi.nlm.nih.gov/pmc/articles/PMC4115576/)
